# Single Cell Quantification of Reporter Gene Expression in Live Adult *Caenorhabditis elegans* Reveals Reproducible Cell-Specific Expression Patterns and Underlying Biological Variation

**DOI:** 10.1371/journal.pone.0124289

**Published:** 2015-05-06

**Authors:** Alexander R. Mendenhall, Patricia M. Tedesco, Bryan Sands, Thomas E. Johnson, Roger Brent

**Affiliations:** 1 Division of Basic Sciences, Fred Hutchinson Cancer Research Center, Seattle, Washington, United States of America; 2 Institute for Behavioral Genetics, University of Colorado, Boulder, Colorado, United States of America; 3 Department of Integrative Physiology, University of Colorado, Boulder, Colorado, United States of America; 4 Biofrontiers Institute, University of Colorado, Boulder, Colorado, United States of America; Inserm U869, FRANCE

## Abstract

In multicellular organisms such as *Caenorhabditis elegans*, differences in complex phenotypes such as lifespan correlate with the level of expression of particular engineered reporter genes. In single celled organisms, quantitative understanding of responses to extracellular signals and of cell-to-cell variation in responses has depended on precise measurement of reporter gene expression. Here, we developed microscope-based methods to quantify reporter gene expression in cells of *Caenorhabditis elegans* with low measurement error. We then quantified expression in strains that carried different configurations of *P_hsp-16.2_-fluorescent-protein* reporters, in whole animals, and in all 20 cells of the intestine tissue, which is responsible for most of the fluorescent signal. Some animals bore more recently developed single copy *P_hsp-16.2_* reporters integrated at defined chromosomal sites, others, “classical” multicopy reporter gene arrays integrated at random sites. At the level of whole animals, variation in gene expression was similar: strains with single copy reporters showed the same amount of animal-to-animal variation as strains with multicopy reporters. At the level of cells, in animals with single copy reporters, the pattern of expression in cells within the tissue was highly stereotyped. In animals with multicopy reporters, the cell-specific expression pattern was also stereotyped, but distinct, and somewhat more variable. Our methods are rapid and gentle enough to allow quantification of expression in the same cells of an animal at different times during adult life. They should allow investigators to use changes in reporter expression in single cells in tissues as quantitative phenotypes, and link those to molecular differences. Moreover, by diminishing measurement error, they should make possible dissection of the causes of the remaining, real, variation in expression. Understanding such variation should help reveal its contribution to differences in complex phenotypic outcomes in multicellular organisms.

## Introduction

Genetically identical organisms grown in homogeneous environments nonetheless show considerable variation in quantitative phenotypes. This is true for bacteriophages (e.g., burst size [1]), bacteria, (e.g., chemotaxis [2]), and yeast (e.g., gene expression and cell signaling [3]). It is also true for isogenic multicellular organisms, for example *Caenorhabditis elegans* (e.g., lifespan [4,5]) and mice (e.g., mass of kidneys [6]), and monozygotic human twins raised together (e.g., measures of physical strength [7] and lifespan [8]). In most cases, the sources and molecular explanations for such variation remain unclear.

In previous work, we identified and quantified sources of variation in quantitative phenotypes defined by amounts of gene expression in *Saccharomyces cerevisiae* [3]. We used reporter genes to measure different sources of variation in gene expression in yeast (stochastic variation in gene expression, variation in gene expression capacity, and variation in signaling to the gene's promoter). These differences can be consequential, for example, yeast cells that have higher gene expression capacity express proteins at a higher rate and increase in volume more rapidly. In those studies, our ability to measure cell-to-cell variation in expression phenotype and to quantify the different contributions to it depended on methods developed to minimize sources of variation in the measurements themselves [9]. Here, we carried out similar work to enable quantification of different sources of variation in the expression of reporter genes in a multicellular organism, *C*. *elegans*.

The use of reporter genes to gain biological knowledge, for example as in [3], has always relied on the ability to construct well-behaved reporters and quantify their expression (see review of reporter genes in S1 Text, Section 2). In *C*. *elegans*, methods for making reporter genes have historically been unique to this organism (see review of *C*. *elegans* transgenesis in S1 Text, Section 3). Expression of canonical multicopy reporters can be erratic (see review of regulation of repetitive DNA in S1 Text, Section 4). However, the recent advent of MosSCI and Cas9 based technologies in *C*. *elegans* has allowed scientists to control transgene locus and copy number (additional details in S1 Text, Section 3).

We previously studied reporters whose expression correlates with lifespan. We studied expression from animals bearing an integrated multicopy *Phsp-16*.*2::gfp* reporter (here written *P*_*hsp-16*.*2*_ *-GFP*) that fused the promoter for this small heat shock protein to the coding sequence of *Aequorea victoria* Green Fluorescent Protein (GFP). We gave young adult animals a heat shock and measured whole animal expression by green fluorescence signal from the *P*_*hsp-16*.*2*_ *-GFP* reporter in flow. These, and subsequent studies with additional single copy *P*_*hsp-16*.*2*_ *-GFP* reporter strains, showed that young adult animals that expressed high amounts of GFP lived longer [10,11]. The mechanistic relationship between the two measured variables, reporter expression and lifespan, remains unclear.

Here, to better understand the relationship between *P*_*hsp-16*.*2*_ reporter configuration and variation in reporter gene expression, we quantified reporter expression in strains that carried reporters with different copy numbers integrated at different loci. We measured expression of different *P*_*hsp-16*.*2*_ reporter strains in whole worms in flow. In flow, strains with higher reporter copy number showed increased fluorescent signal. The relationship between expression and copy number was linear at low copy number and nonlinear at high copy number. We observed no difference in worm-to-worm variation in reporter expression among these strains.

To measure cell-to-cell variation in gene expression we developed methods to measure reporter expression in individual cells in live adult animals via microscopy. Because approximately 90% of the reporter signal in adult animals expressing *P*_*hsp-16*.*2*_ *-GFP* comes from the 20 cells of the intestine [11,12] (additional details in Results and S1 Text, Section 1), we developed low measurement error methods to measure reporter gene expression in these cells. Both a characterized 530 copy reporter strain and a single copy strain showed consistent patterns of expression. However, these cell-specific expression patterns were quite distinct; for example, each reporter was consistently expressed most highly in different intestine cells.

Our development of precise single cell reporter measurement methods should allow investigators to use real-time changes in reporter expression in cells in functional tissues as quantitative phenotypes in small-scale genetic screens in live animals. The methods minimize the amount of time the animals need to be imaged and leave the animals alive, physically intact and functional, allowing subsequent monitoring of the same cells in longitudinal studies of single cells. These methods should enable the use of fluorescent protein reporters to reveal real differences in quantitative physiology of individual cells in tissues, and allow dissection of sources of cell-to-cell variation in gene expression. Understanding variation in cell-specific gene expression should reveal its contribution to corresponding differences in complex phenotypic outcomes in multicellular organisms.

## Materials and Methods

We present key methods details in Results and provide additional, more detailed information in Supporting Information (S1 Text, Section 5).

### Strains, Culture and Measurement Conditions

**Fig 1:**
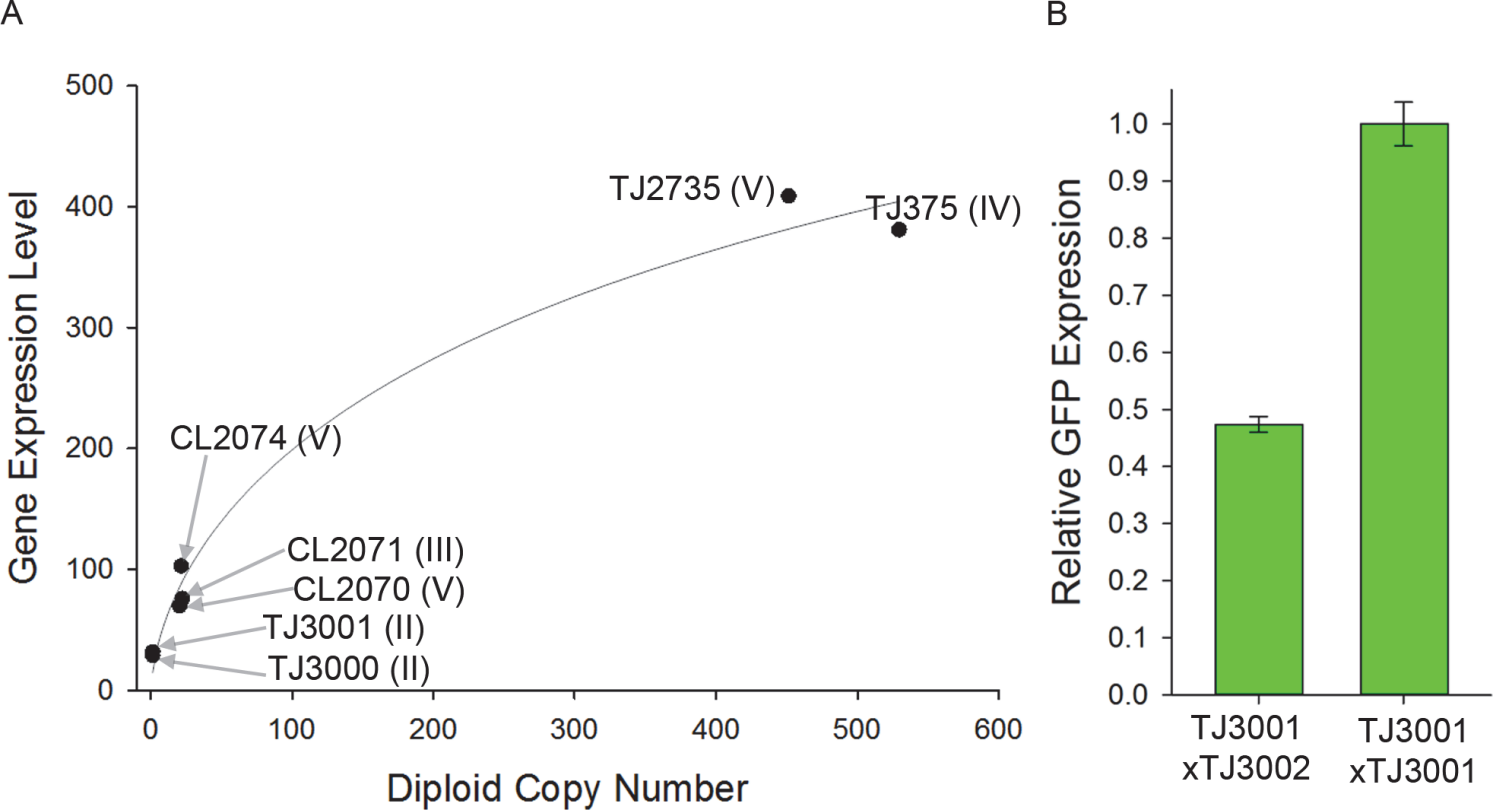
Relationship between copy number and expression in *P_hsp-16.2_*-XFP reporter strains. **A)** X axis is reporter diploid copy number; Y axis shows expression level in PMT counts. Solid line shows a Hill function with a Hill coefficient of 0.6 (a quantitative measure of the nonlinear increase in expression with increasing copy number), fit to the data with an R^2^ of 0.97. For each strain, the expression data is the average of at least three flow experiments that quantified about 500 animals per experiment. **B)** Average expression level of a single copy *P_hsp-16.2_* *-GFP* reporter in homozygotes and heterozygotes. Error bars show S.E.M. We picked over 200 F1 hermaphrodites from each cross and measured 84 F1 heterozygotes and 49 F1 homozygotes in flow; additional details in S1 Text, Section 5: Strain Construction.

Table 1 shows strains. Three: CL2070, CL2071 and CL2074, carried "complex" multicopy arrays; four: TJ375, TJ2732, TJ2733 and TJ2735, carried repetitive multicopy arrays; four: TJ3000, TJ3001, TJ3002 and RBW2, carried single copy *P*_*hsp-16*.*2*_ *-fluorescent-protein* (hereafter, *P*_*hsp-16*._-*XFP* reporters) integrated at a defined site in chromosome II. RBW2 also contained a biolistically integrated transgene, not on chromosome II. RBW2642 and RBW2661 contained single copy *P*_*daf-21*_ *-XFP* reporters at a defined site in chromosome II. Vector construction, transgenesis and copy number measures are detailed in S1 Text, Section 5. For the experiments in Fig 1, we generated F1 progeny of a TJ3001 x TJ3002 cross; we also used such F1 progeny in experiments measuring variation in gene expression in the last section of the Results. TJ2732 (*P*_*hsp-16*.*2*_ *-dsRed)* and TJ375 (*P*_*hsp-16*.*2*_ *-GFP)* animals were also bred together to make a two-reporter strain for experiments measuring variation in gene expression in the last section of the Results. We grew animals at 20°. In all experiments, we synchronized strains via alkaline hypochlorite treatment [13], grew them for 72 hours at 20°, heat shocked them for one hour at 35°, allowed them to recover for 24 hours at 20°, then quantified reporter expression in flow or microscopically as described in [11].

**Table 1:** Reporter Strains.

Strain	Reporter	Copy #^a^	Chr.
TJ375	*P*_*hsp-16*.*2*_::*gfp::T*_*unc-54*_	530	IV
TJ2735	*P*_*hsp-16*.*2*_::*gfp*:: *T*_*unc-54*_	450	V
TJ2732	*P*_*hsp-16*.*2*_::*dsred::T*_*let-858*_	500	V
TJ2733	*P*_*hsp-16*.*2*_::*dsred*:: *T*_*let-858*_	940	IV
CL2070	*P*_*hsp-16*.*2*_::*gfp*:: *T*_*unc-54*_ *;rol-6(su1006)*	20	V
CL2071	*P*_*hsp-16*.*2*_::*gfp*:: *T*_*unc-54*_ *;rol-6(su1006)*	22	III
CL2074	*P*_*hsp-16*.*2*_::*gfp*:: *T*_*unc-54*_ *;rol-6(su1006)*	21	V
TJ3000	*P*_*hsp-16*.*2*_::*gfp*:: *T*_*unc-54*_ *;cb-unc-119(+)*	1	II
TJ3001	*P*_*hsp-16*.*2*_::*gfp*:: *T*_*unc-54*_ *;cb-unc-119(+)*	1	II
TJ3002	*P*_*hsp-16*.*2*_::*mcherry*:: *T*_*unc-54*_ *;cb-unc-119(+)* (II)	1	II
RBW2	*P*_*hsp-16*.*2*_::*mcherry*:: *T*_*unc-54*_ *;cb-unc-119(+)* (II);*P*_*lmn-1*_::*emr-1::gfp::T*_*lmn-1*_ (not II)	1,?	II; not II
RBW2661	*P*_*daf-21*_::*gfp::T*_*unc-54*_ *;cb-unc-119(+)*	1	II
RBW2642	*P*_*daf-21*_::*mcherry::T*_*unc-54*_ *;cb-unc-119(+)*	1	II

a—Copy number was determined via qPCR, using *ama-1* as a control, detailed in S1 Text, Section 5.

### Measurement in Flow

We measured GFP or dsRed fluorescence in flow approximately 24 hours after heat shock using a COPAS Biosort instrument (Union Biometrica, Boston). We typically placed about 1000 animals in a 10 mL volume of S-basal into the sample cup and initiated flow to measure approximately 500 animals from each population. In each run, we measured all strains that expressed the same fluorescent protein with the instrument set to the same laser power and gain. We prevented cross contamination between each sample run by cleaning the sample cup and performing three pulses of high pressure to dislodge debris, and monitoring flow after cleaning to confirm the absence of animals.

### Microscopy and Image cytometry

#### Preparation of animals for microscopy

We mounted animals on 1% agarose pads on microscope slides and anesthetized them gently with a tricaine/tetramisole solution as in [14] to eliminate/minimize movement. Before imaging, in order to allow the anesthetic to take effect, we placed the slides on a plastic rack one centimeter above an ice bath of approximately 2 liters in volume (2 liters of crushed ice in an 8 inch x 12 inch plastic box) for about an hour. This treatment cooled the slide from room temperature and maintained it at 12–16° for a few hours (during the course of imaging). The fact that animals were still alive and moving after imaging sessions showed that anesthesia and imaging conditions were fairly gentle. Animals could be recovered from slides, though they were not in this particular study. The usual alternative anesthetic, sodium azide, has more negative side effects such as damaging the animals’ tissues, inducing expression of chaperones including *hsp-16*.*2* [15], and eventually killing the animal.

#### Image acquisition for single cell cytometry

We used an inverted Zeiss LSM 510 point-scanning confocal microscope with a 20X 0.8 NA objective to image animals. We used a 488nm laser to excite the sample and a 505–550 nm bandpass emission filter to collect the emitted light. For the 10X dimmer TJ3001 animals bearing the single copy reporter, we kept the laser power to the sample the same, but increased the gain of the photomultiplier tube. We took 2 micron steps in z and captured confocal slices with an optical slice thickness of 1.8 microns (about one Airy unit with a 20x 0.8 NA air objective). We took 15–30 Z slices in each field of view (2–3 fields per worm) to ensure we captured all of the nuclei in all of the cells in the 50 micron diameter worm body. For measures of signal attenuation with depth, emission spectrum quantification, FRAP, and imaging comparisons of the nucleus and the cytoplasm fluorescent signal, we used a 40X, 1.3 NA objective on a Zeiss LSM 780, and to image the mCherry signal, we used a 561 nm excitation laser and collected emitted light from 569–694 nm using a spectral detector array (an array of highly sensitive GaAsP-photomultiplier tubes aligned behind a diffraction grating). Images of animals expressing two different *P*_*hsp-16*.*2*_ reporters at the same time were acquired on a Zeiss epifluorescent microscope with a 40X, 1.3 NA objective.

#### Quantification of reporter expression from cell images

**Fig 2:**
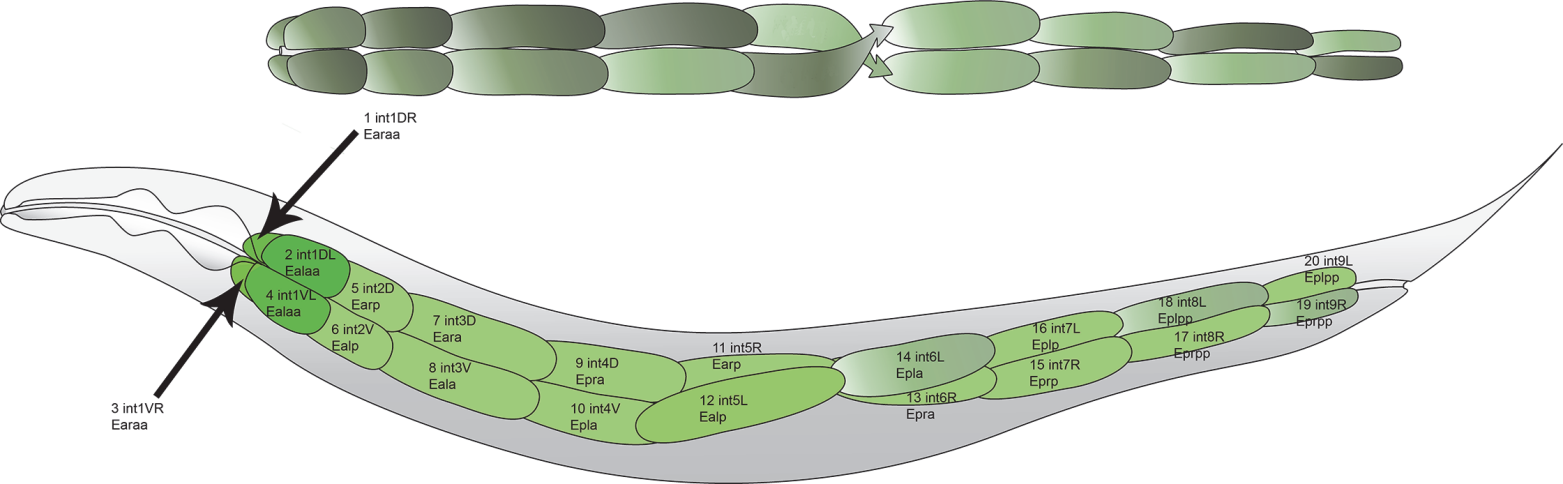
Layout of cells comprising the adult *C*. *elegans* intestine. Top panel shows the location of the left-handed helical half twist. Bottom panel shows the intestine within an animal. Anteriormost ring is comprised of four cells, remaining rings are made up of two cells, all arranged around a hollow core with the half twist between ring V and ring VI. Cells are identified by proper intestine cell name (e.g., int5L), progenitor cell (lineage, e.g., Ealp), and number (e.g., #12). Cells in L lineage are even numbers and cells in R lineage are odd numbers. We refer to these cells by their numbers in the x axis of Fig 4. A more anatomically detailed cartoon with corresponding microscopic images that are also anatomically detailed is available as S1 Fig; identification of the nuclei in the cells in the twist in microscopic images is also shown in S6 Fig.

**Fig 3:**
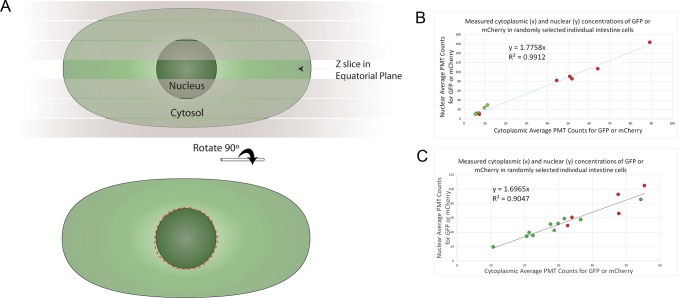
Quantification of single-cell expression by nuclear fluorescence signal. **A)** For each cell, from a confocal stack, we chose the z slice with the largest nuclear perimeter, then, using ImageJ, drew the nuclear boundary using the ellipse tool, and then used the selection brush tool to correct the boundary (approximated here by the red dashed line). We took the average pixel intensity inside these boundaries. Additional detail is shown in S7 Fig, which shows the proper identification of the nuclear equatorial plane in actual confocal micrographs. **B)** Relationship between cytoplasmic and nuclear concentration of fluorescent proteins in intestine cells. GFP or mCherry expression was driven by the *daf-21* promoter in strains RBW2661 (*P_daf-21_-GFP*) and RBW2642 (*P_daf-21_-mCherry*), respectively. Cytoplasmic and nuclear concentrations of fluorescent protein were measured and plotted as x,y coordinates. Red dots are from mCherry expressing cells; green dots are from GFP expressing cells. We measured the nucleus as in **A.** To sample the cytoplasm, we measured a nucleus sized area of the cytoplasm in the same z slice as the equatorial plane of the nucleus, with care to avoid autofluorescent granules in the cytoplasm (S3 Fig). Three randomly selected intestine cells were measured in each of four different animals (12 cells total), two expressing GFP and two expressing mCherry. **C)** The relationship between cytoplasmic and nuclear concentration of fluorescent proteins in intestine cells of heat shocked animals is shown. GFP or mCherry expression was driven by the *hsp-16*.*2* promoter in TJ3001 (*P*_*hsp-16*.*2*_-*GFP*) or TJ3002 (*P*_*hsp-16*.*2*_-*mCherry*) animals, respectively. Measurements were made as in **B**, but cytoplasmic measures were difficult because there was more autofluorescence in heat shocked animals’ cells, and we had to sample a few more cells (15 cells total) to see a decent correlation due to this “noise” in the cytoplasmic measurements. *P* < 10^–7^ for the correlations in **B** and **C**.

We learned to determine the identity of individual cells in the adult worm intestine as described below (Results, S1 Text, Sections 5 and 7, Figs 2, S1 and S6). We used ImageJ [16] to aid manual segmentation of the images and then to extract quantitative information from image files as described (Results, S1 Text, Sections 5 and 7, Figs 3 and S7). We did not correct for photobleaching as we had measured it to be relatively insignificant (~0.3% signal loss measured after taking a second series of 25 z slices and measuring the same slice again) and we only extracted values from samples that had been scanned once. To validate the use of nuclear-enriched GFP to mark the nuclear boundary (see Results), we also used RBW2 animals, which carried a single copy *P*_*hsp-16*.*2*_ *-mCherry* reporter and a gene encoding an Emerin-GFP fusion protein, which is incorporated into the inner nuclear envelope. In images of intestinal cells of these animals, red fluorescent signal was concentrated in elliptical features we identified as the nuclei. The boundary of these features defined by mCherry enrichment coincided with the nuclear boundary identified by Emerin-GFP fluorescence.

#### Statistical analysis of expression data

We used Sigma Stat (Systat Software, Inc., San Jose) for statistical analyses. We first determined if measurements comprising each dataset were normally distributed, then used appropriate parametric or non-parametric statistics to determine if there was significant difference in average expression level or average coefficient of variation (additional details in S1 Text, Section 5). The coefficient of variation (CV, the standard deviation divided by the mean, sometimes termed “relative standard deviation”) is a normalized measure of variation, which allows comparison of distributions of values independent of their means (applicability to quantitative phenotypes reviewed by Lewontin in 1966 [17]). We present each statistical finding in the Results, and compile the results of all the individual tests in S2 Table.

## Results

### Variation in whole animal expression in strains with different reporter constructs is similar

After heat shock, we used a worm sorter (COPAS Biosort, Union Biometrica, Boston; additional details in S1 Text, Section 5) to measure *P*_*hsp-16*.*2*_ *-GFP* and *P*_*hsp-16*.*2*_ *-dsRed* expression in the strains in Table 1. We did this in two series of experiments ("campaigns"). Campaign 1 quantified expression of all multicopy reporter strains together, in each of five separate runs. Campaign 2 quantified, in 10 separate runs, expression in a single copy strain, TJ3001, and in strain TJ375, which contained the well-studied 530 copy repetitive array [10]. We used a normalized measure of variation, the Coefficient of Variation (CV = Standard Deviation / Mean), often used to compare variation in distributions with different means (reviewed by Lewontin [17]). There was no correlation between average *P*_*hsp-16*.*2*_ *-XFP* (dsRed or GFP) expression and worm-to-worm variation in expression, nor between copy number and variation in expression (R^2^ < 0.1; *P* > 0.4) (S1 and S2 Tables). Moreover, there were no differences in worm-to-worm variation in expression attributable to other differences in the reporter strains, including identity of XFP (GFP or dsRed), identity of 3’UTR (*unc-54* or *let-858*), residence in "simple" or "complex" arrays, or residence on a particular chromosome (S2 Table; array types defined in S1 Text, Section 3).

### Reporter expression is only proportional to copy number at low copy numbers

Fig 1A shows expression levels of seven strains (1–530 copies of *P*_*hsp-16*.*2*_ *-GFP* reporters) in flow. We also measured expression in strains heterozygous and homozygous for the zSi3001 reporter allele in the TJ3001 strain. Fig 1B shows that signal from the homozygote (*P*_*hsp-16*.*2*_ *-GFP* reporter on each copy of chromosome II) was twice that of F1 heterozygotes (*P*_*hsp-16*.*2*_ *-GFP* reporter on only 1 copy of chromosome II). Increased GFP expression per additional gene copy diminished as copy number increased: strains with 20 copies had about three times the GFP signal as single copy strains, strains with ~500 copies about ten times. For strains whose reporter number varied from 1 copy to 530, dependence of expression on copy number was overall monotonic, but showed less than linear increase. The less than linear increase in gene expression with copy number fit a Hill function with Hill coefficient of 0.6. We note that two strains that carried ~20 copy arrays (CL2070 and CL2074), products of different integration events on the same chromosome, differed in *P*_*hsp-16*.*2*_ *-GFP* expression (70 vs 100 average PMT counts; S1 Table; *P* = 0.04; Paired t-test).

### Careful examination of confocal images permits identification of individual intestine cells

We quantified expression of *P*_*hsp-16*.*2*_ *-GFP* in individual cells. We did so in order to compare expression in the different cells of an individual animal's intestine, and to compare expression in the same cells in different animals. To do so, we needed to determine the orientation of each animal and to learn to identify the individual cells in the adult from confocal micrographs. Orienting the animal by finding the head (anterior pole) and vulva (ventral pole) was straightforward. Identifying specific intestine cells in the adult intestine by cell name and lineage relationship had not been done previously. This identification presented difficulties because the intestine crosses from left side to right side at ring V, and thus, for worms lying on their sides, changes position in z. Moreover, the egg-filled uterus squeezes and distorts the intestine at the junction between ring V and ring VI, while, at the same place, the cells of the intestine form a left-handed helical half-twist (Figs 2, S1 and S6), presumably the twist observed by Sulston in the L1 larva [18].

Cartoons in Figs 2 and S1 show the layout of intestine cells in the adult; and the bottom two panels of S1 Fig show micrographs of those same cells in a TJ3001 animal expressing a single copy *P*_*hsp-16*.*2*_ *-GFP*. Cells in ring I through ring IV are named Dorsal, D and Ventral, V (e.g., int2D), and cells in ring I are further designated Left, L, and Right, R (e.g., int1DL). Cells in ring V through ring IX are named *based on their position in the embryo* as Left, L and Right, R (e.g., int6R). During embryonic development at 20°, at some point later than 430 minutes post fertilization [19] and before hatching [18], the embryonic Left and Right cells in the three posterior rings become respectively Dorsal and Ventral (cells in rings VII-IX; Personal Communication from Jim Priess), turning the embryonic quarter-twist [19] into the left-handed helical half-twist found in larvae [18]. In adult animals, the cells in this helical half-twist were often difficult to identify because of their occlusion in z by another equally large intestine cell, so we typically started cell identification with ring I and worked posteriorly to ring V. We then identified cells in ring IX and worked anteriorly to ring VI. We used the nucleus or nuclei present in each cell to help account for each cell as we worked anteriorly or posteriorly toward the middle of the animal. Cells in ring V and ring VI, where the left-handed helical half-twist occurs, connect to anterior or posterior cells that are clearly dorsal or ventral and not occluded by other intestine cells. Thus, we used the identity of neighboring cells to determine the identity of cells in the left-handed helical half-twist. Specifically, int5L is connected anteriorly to the clearly ventral int4V and posteriorly to int6L. The int6L cell is connected posteriorly to the clearly dorsal int7L. Conversely, int5R is connected anteriorly to clearly dorsal int4D, and posteriorly to int6R, which is connected posteriorly to clearly ventral int7R. To assist other investigators in identifying intestine cells, we deposited this information and images (of RBW2 animals) at www.wormatlas.org. We also provided, in supplementary information, microscopic images showing the layout of the cells in an adult animal (S1 Fig) and showing the identification of all eight nuclei in the four cells comprising the intestinal left-handed helical half-twist (S6 Fig). Moreover, we provide a step by step protocol for imaging animals, identifying all of the intestine cells and nuclei, and quantifying reporter expression in each cell in S1 Text, Section 7.

### Nuclear enrichment of XFP signal enables consistent manual quantification of single cell gene expression

We quantified *P*_*hsp-16*.*2*_ *-GFP* reporter expression in individual intestine cells after we identified them. We took advantage of the nuclear enrichment of XFPs in *C*. *elegan*s cells (Fire Lab Vector Kit documentation 1995; Personal Communication from Andrew Fire) to define the nuclear boundary, and used fluorescence signal from the nucleus as a proxy for whole cell fluorescent signal to work around other sources of measurement variation (discussed below). Although nuclear enrichment of XFPs is widely observed and occasionally commented on in mammalian cells (for example for: "cycle 3 GFP" [20] and eGFP [21,22]), its cause(s) is not understood. We consider possible causes of this nuclear enrichment elsewhere, but note here that the enrichment we see (1.7X - 1.8X; Fig 3) is associated with slower diffusion in *C*. *elegans* intestinal nuclei we measured by FRAP (about 1.7 X slower; S2 Fig) and reported by others [21].

**Fig 4:**
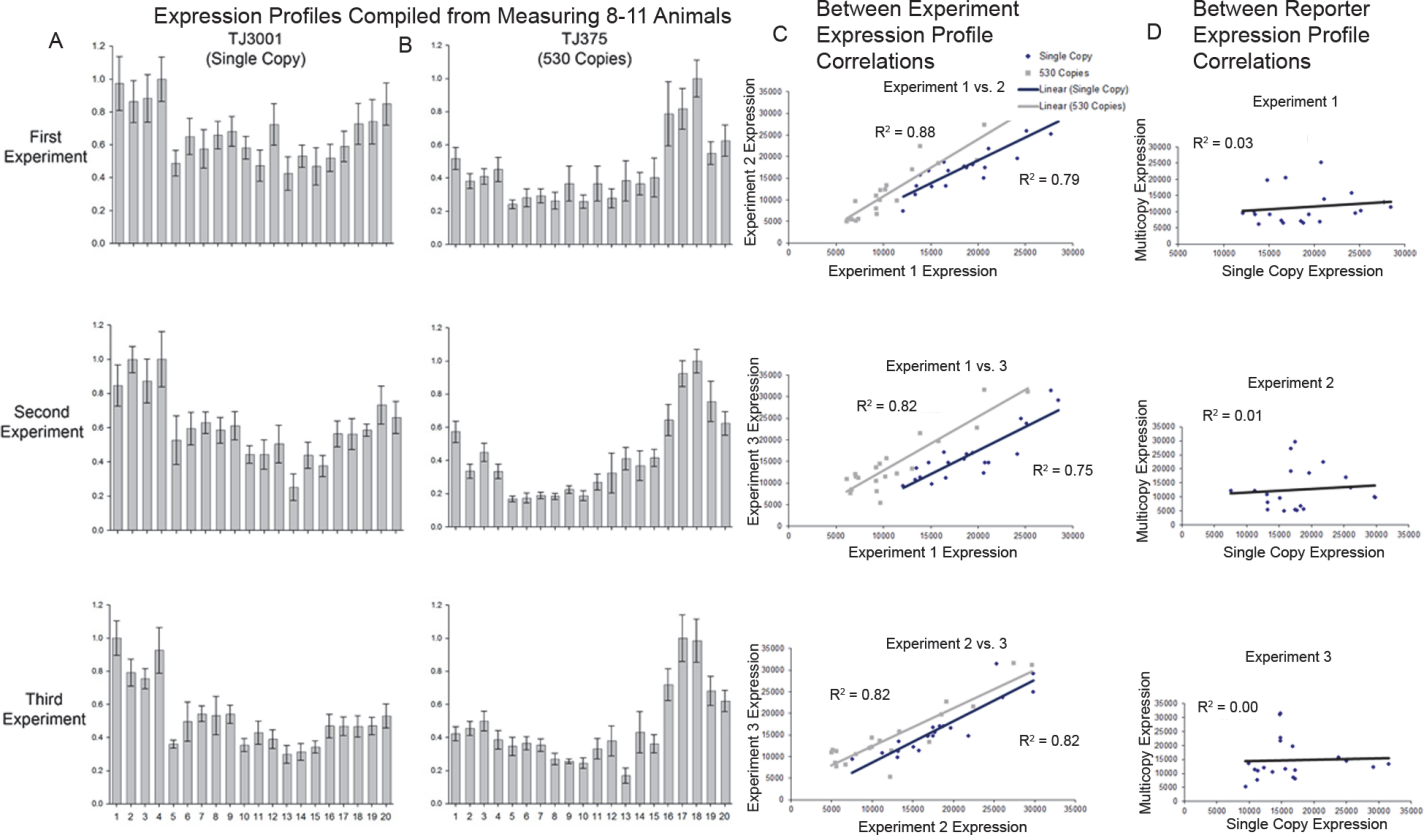
Consistency of experimental measurements. **A & B)** Single-cell expression profiles of different *P*_*hsp-16*.*2*_-*GFP* reporters in the 20 cells of the intestine from three different experiments. The different intestinal cells are laid out anterior to posterior, and numbered 1 to 20. They correspond to the cell names and lineage relationships detailed in Figs 2 and S1. The heights of the bars show expression level normalized to the brightest cell for each reporter in each experiment. **C)** Consistency of cell-specific expression patterns within a strain. Consistency of expression profiles of *P*_*hsp-16*.*2*_-*GFP* reporters in individual cells in TJ3001 (single copy) and TJ375 (530 copy) animals. Results come from three experiments on three different days. Plots show average expression for a given cell from one experiment as x, and average expression for the same cell measured in a different group of animals in a different experiment as y, for all combinations of two experiments. Expression profiles were between 75 and 88% correlated between experiments. **D)** Lack of similarity of cell-specific expression patterns between single copy and 530 copy strains. Average expression values for the single copy reporter were plotted as x, and the average expression value for the same cell in different animals expressing the multicopy reporter was plotted as y. Cell-specific expression from these two different *P*_*hsp-16*.*2*_ reporter strains is not correlated.

To quantify XFP expression once we had assigned an identity to a given cell, we found the z slice of the image file that contained the largest diameter image of its nucleus (Figs 3 and S7) or, if the cell had two nuclei, the individual slices that had the largest diameter image of each one. Next, using ImageJ [16], we manually drew the nuclear perimeter using the oval, ellipse and/or selection brush tools. We then used the ImageJ "measure" function to acquire average pixel intensity inside the manually segmented perimeter (additional details in S1 Text, Section 7). For each image, we repeated this procedure two more times and took the average value from the three trials to control for human error in drawing the boundary during segmentation. For cells that contained two nuclei, we took the average intensity of both. To determine signal above background, we then measured slide background from six areas of the image of approximately the same size and shape as a nucleus, averaged those, and subtracted that average value from final average nuclear value for each cell. Fig 4C shows a measure of the precision given by these methods—the inter-experiment consistency of expression data.

We used fluorescence signal from a single Z slice of the ellipsoidal nucleus as a proxy for whole cell gene expression. We came to this approach after considerable experimentation directed toward diminishing variation in fluorescence measurements. This variation had a number of causes. First, even after learning to determine the identity of the individual cells, we found it difficult to reliably recognize the cytoplasmic boundary between cells, while the nuclear boundary defined by XFP enrichment was relatively easy to find. Second, cytoplasmic signal was highly heterogeneous, due to bright objects, probably secondary lysosomes [23], that emitted an overlapping fluorescent signal with a different spectrum (S3 Fig), and also due to dark objects, probably vesicles, that contained no fluorescent signal (S3A and S3B Fig). By contrast, the nuclear fluorescence signal was relatively homogeneous (S3 Fig). Even nuclear images from z slices containing the nucleolus (which lacked signal) showed less signal heterogeneity than corresponding images of the cytoplasm (S3A, S3B and S3C Fig). Third, the longer we imaged an animal, the more we photobleached our signal and perturbed the animal (e.g., by being anesthetized, deprived of food and scanned by a laser). Capturing the single z slice that contained the equatorial plane of the nuclear image allowed us to spend less time subjecting cells in the animal to laser illumination, and reduced diminution of signal. Reducing imaging time may have also reduced other changes in signal due to changes in cell physiology caused by photochemical damage by the laser light, prolonged exposure of the organism to anesthesia, or prolonged residence of the organism without food on an agarose pad. Fourth, fluorescent signal diminished significantly with increasing distance of the z slice from the microscope objective (S4 Fig), making difference in depth a source of measurement error. Variation from the third and fourth sources argued against an alternative approach of estimating fluorescence from the entire nuclear volume by reconstituting fluorescence signal from all the images of a given nucleus in the same z stack.

### Quantification of fluorescence signal shows consistent but distinct expression patterns for each reporter

We measured expression in all 20 intestinal cells from 28 single copy TJ3001 animals (3 experiments, 8–10 animals each) and 31 530 copy TJ375 animals (3 experiments, 10–11 animals each).

**Fig 5:**
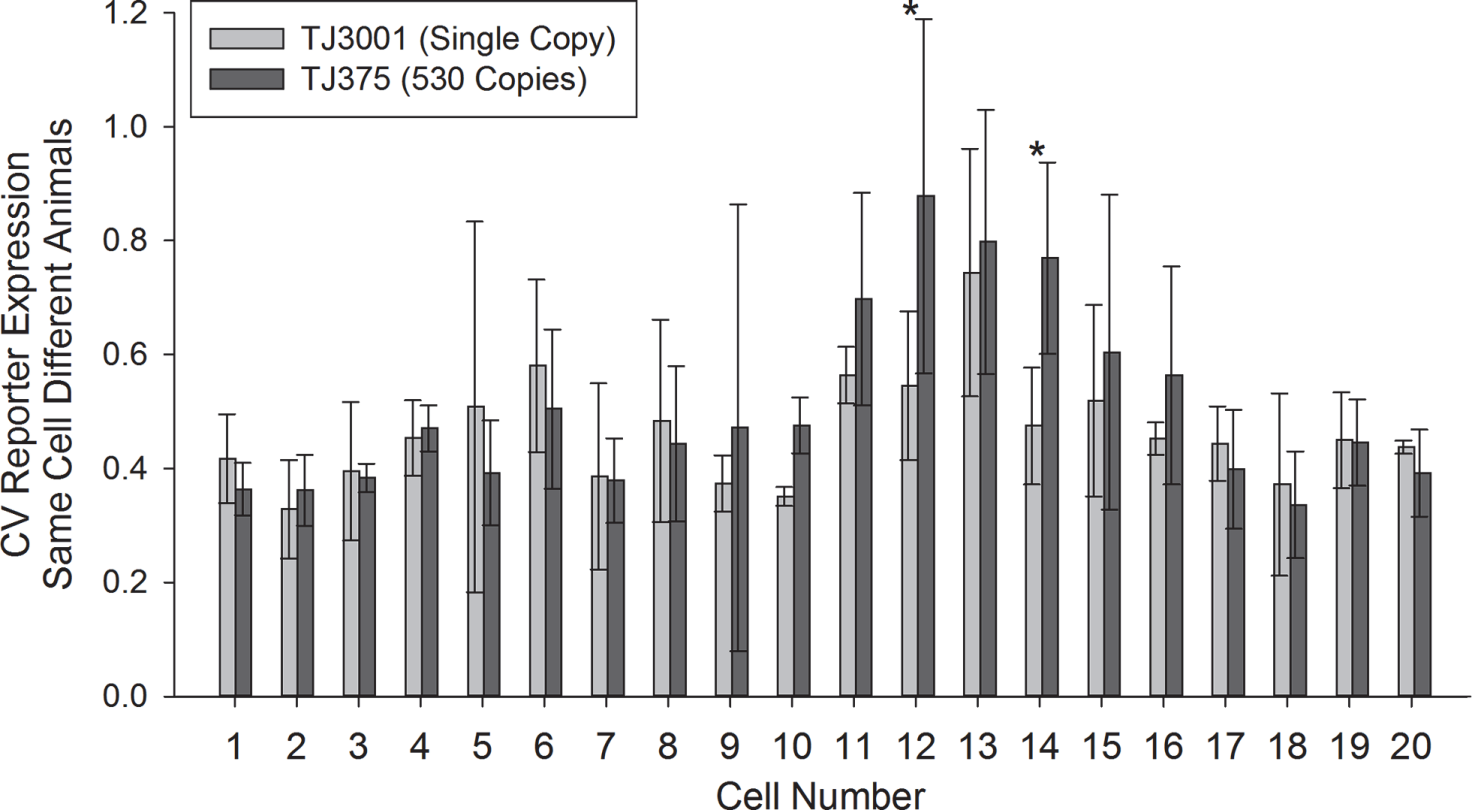
Variation in expression among the same cells in different animals. Plot shows variation in *P*_*hsp-16*.*2*_-*GFP* expression for each of the twenty intestine cells by image cytometry (3 CV values per cell, with each CV derived from 8–11 measures). X axis shows cell identity (detailed in Figs 2 and S1). Bars show average coefficient of variation. Error bars show standard deviation. Stars above bars indicate significant differences in CV (*P* < 0.022; Two-Way Repeated Measures ANOVA followed by a Holm-Sidak comparison procedure) between the two reporters in Cell 12 (int5L) and Cell 14 (int6L).

Fig 4 shows the results. For each strain, the cell-specific pattern of *P*_*hsp-16*.*2*_ *-GFP* expression within the tissue was consistent among different individuals (75%-88% correlation, Fig 4A–4C). However, compared to the single copy strain, cell-specific expression for the 530 copy strain was quite different (Fig 4A, 4B and 4D). For example in TJ3001, the single copy reporter strain, the four cells of ring I, derived from the anterior gut lineage (E_a_), gave the highest signal, while in TJ375, the 530 copy strain, the two cells of ring VIII, from the posterior gut lineage (E_p_), expressed the most signal (Fig 4A and 4B). We then quantified cell-to-cell variation of the different reporters in the same cell, but among different animals (e.g., cell #1, int1DR). Fig 5 shows the results. Most intestine cells had a CV for *P*_*hsp-16*.*2*_ *-GFP* expression between 40–60%. Variation in *P*_*hsp-16*.*2*_ *-GFP* expression for the same cell among different animals was higher for two cells in animals bearing the 530 copy array (*P* < 0.022; in int5L and int6L; Cells 12 and 14 in Fig 5). By contrast, no cell in the single copy strain TJ3001 showed more same-cell-different-animal variation in *P*_*hsp-16*.*2*_ reporter expression than its counterpart in TJ375 animals.

To compare these measurements to those we obtained from whole animal fluorescence measured in flow above, we summed *P*_*hsp-16*.*2*_ *-GFP* expression from the 20 individual intestine cells, to generate a total expression value for each animal. As determined by image cytometry, animal-to-animal variation (as measured by the CV) in total intestine expression of *P*_*hsp-16*.*2*_ *-GFP* in the single copy and 530 copy strains was almost the same, and matched the values measured in flow (no significant differences; Table 2). The lack of difference between expression variation measured in flow and by microscopy further validated the use nuclear fluorescence as a proxy for whole cell fluorescence.

**Table 2:** Interindividual variation in expression of *P*_*hsp-16*.*2*_-*GFP* reporter strains by flow and by image cytometry.

*P*_*hsp-16*.*2*_ Reporter	Mean Flow CV^a^	Mean Microscope CV^b^	Mean Expression^c^
Single Copy^d^	23.0	21.9	32
530 Copy Repetitive Array^e^	21.8	21.3	310

a—For flow measurements, numbers come from about 500 animals measured for each strain, repeated in ten runs on ten different days. CV is the Coefficient of Variation, or relative standard deviation (CV = Standard Deviation/Mean).

b—For microscopic measurements numbers come from the summed values of all intestine cells measured with image cytometry, which is a total of three measured CV values for each reporter.

c—Mean expression measured in flow. We could not compare mean expression data acquired by microscopy because we needed to increase the PMT detector gain for the single copy reporter.

d—Strain TJ3001.

e—Strain TJ375.

### Cell-to-cell variation in *P*_*hsp-16*.*2*_ reporter expression from multicopy arrays is different from variation in expression from single copy reporters

In part to facilitate future studies of sources of stochastic noise in gene expression [3,24] we made animals in which we could measure the output of two different instances of the same promoter in the same cell. To do so, we generated a strain that carried multiple copy *P*_*hsp-16*.*2*_ *-dsRed* and *P*_*hsp-16*.*2*_ *-GFP* reporters on autosomes IV and V, and also generated F1 animals (F1 progeny of TJ3001 x TJ3002 cross; Fig 1B) that carried single copies of *P*_*hsp-16*.*2*_-mCherry and *P*_*hsp-16*.*2*_ *-* GFP reporters at the same site on each copy of chromosome II.

**Fig 6:**
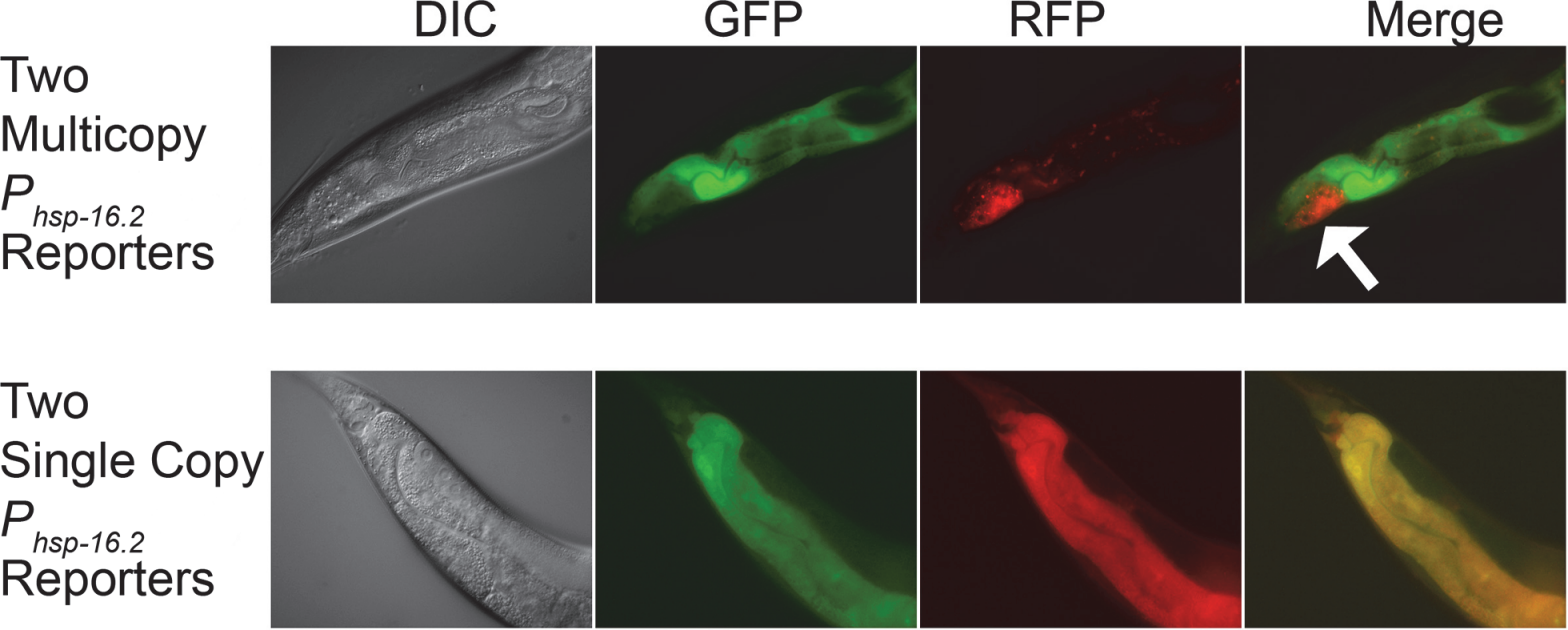
Expression of two differently colored *P*_*hsp-16*.*2*_ reporters in the same animal using multicopy and single copy reporters. DIC micrographs and fluorescent images show the three posterior-most intestinal cell rings (rings VII-IX). In the figure, posterior is on the left and dorsal on the top. Top row shows an animal expressing two multicopy reporters. Bottom row shows an animal expressing two single copy reporters. In about 1/3 of the animals expressing two multicopy reporters, there is a dramatic difference in the expression of the reporters in at least one cell. The strong bias toward expression of the red allele in int9R (white arrow, top row, fourth panel) occurred in slightly more than half of the animals showing differential expression (12/21). We did not observe such pronounced expression bias in the intestine cells of the animals expressing the two single copy reporters (typical image shown in bottom row).

When we imaged the two differently colored (GFP and mCherry) single copy reporters expressed together in the same animal, we observed uniformly similar red and green output in individual intestine cells (Fig 6 bottom row). However, Fig 6 shows a surprising pattern of expression for the animals bearing the two multicopy arrays. In about one third of the animals (34.2 +/- 5.6%; two experiments), at least one cell showed strong expression bias for expression of one reporter or the other. Within this group (which was selectable by eye using a fluorescent stereoscope), most (12/21) of the animals expressed the dsRed reporter allele in Int9R (Fig 6 top row), and the rest showed strong expression bias in other cells and/or for the GFP reporter allele. This result shows that, for this reporter, expression of multicopy reporters is subject to additional sources of variation of unknown origin (see Discussion, and also S1 Text, Section 4) that could confound attempts to measure stochastic noise in gene expression.

## Discussion

### Precise quantification of reporter expression

We developed precise means to quantify expression of fluorescent protein reporter genes in each of the cells in an entire *C*. *elegans* tissue, the intestine. We did so by learning to identify each cell, by using defined-site, single copy reporters, and by systematically reducing or circumventing additional sources of variation that would otherwise be introduced during the process of measurement. We used the nuclear fluorescence signal from a single consistently determined confocal slice as a proxy for the signal from the entire cell. Doing so enabled us to segment the images and quantify fluorescent signal consistently from experiment to experiment.

### Comparison with other methods of measurement to quantify XFP expression in *C*. *elegans*

Other reports [25,26], including our prior work [10,11], measured *C*. *elegans* and fluorescent signals from reporter genes in flow. In particular, relatively recent work using a newer model worm sorter used XFP reporters to monitor expression from many different promoters, finding different promoter-specific patterns of gene expression along the length of the worm [25]. By contrast our work in flow only examined total fluorescence from individual animals and our methods development focused on eliminating experimental error, in this case due to cross contamination of specific samples.

At least three prior studies used fluorescent protein signal to monitor gene expression in large numbers of individual *C*. *elegans* cells. In 2009, Liu et al. used a program called VANO [27] to automatically identify individual cells’ nuclei and quantify single-cell reporter expression in fixed, killed L1 larvae [28]. The program used a DAPI stain to identify each nucleus, and signal from a Histone H1.1-mCherry derivative (*his-24::mCherry*; incorporated into chromatin as a linker between nucleosomes), expressed from transgenes of unknown locus and copy number, to quantify expression driven by 93 different promoters. By contrast, the methods developed here monitored expression in cells of living, adult animals. Expression was from single copy reporters integrated at defined sites, and our reporter gene product was not incorporated into chromatin, but rather, freely diffusing within cells. Our methods do not use computer aided cell identification or automated quantification. Our throughput is therefore appropriate for genetic screens testing the effects of candidate genes but not for genome wide screens.

Two studies from a different group developed and used sophisticated means to monitor cell-specific gene expression in living embryos [29,30]. Both quantified reporter expression in confocal images of individual embryos up to the 350-cell stage, when the embryo starts moving. In the first study [29], to mark the nuclei, the researchers used signal from two different GFP transgenes whose products are incorporated into the core nucleosome, a *P*_*pie1*_ *-H2B-GFP* gene (*Ppie-1::his-11::gfp::Tpie-1*) and an H3.3-GFP (*his-72::gfp*) gene. To monitor expression from different promoters, they used transgenes in which those promoters directed expression of H1.1-mCherry and H1.1-dsRed (*his-24::mCherry* and *his-24::dsRed*). Such H1.1-XFP fusion proteins are integrated into chromatin and exchange with native H1.1 [31]. H1.1 helps keep DNA wrapped around the core nucleosome and also binds the intercore linker DNA (reviewed in [32]). In *C*. *elegans*, H1.1 is required for germline silencing [33], where it appears to be sequestered into cytoplasmic structures whose other components and function are not well understood [34]. Using these fluorescent reporter proteins, Murray et al. identified, and then quantified, fluorescence signal from individual nuclei. To do so, they used two programs. One, Starry Night [35], analyzed image time series and output files that described each nucleus’s location and lineage relationship. Another, AceTree [36], a companion GUI, displayed lineage relationships and facilitated annotation and curation. In a second study [30], the same researchers used these methods to monitor whole-embryo expression of 127 different embryonically expressed genes. In this later study, to monitor expression, the authors used promoters fused to histone-GFP and histone-mCherry derivatives as in the 2008 study, and also an additional group of GFP transcription factor fusion proteins, and tracked cells with a H1.1-mCherry fusion protein driven by the H3.3 (*his-72)* promoter.

By contrast, although our methods should work on cells of transparent organisms, including embryos, in this study we quantified gene expression in cells of the adult animal. An important distinction between the methods described by Murray et al. and this work is that our methods are much less sophisticated. Our methods require significant human effort, to identify the cells, to delineate the nuclear boundary, and to measure signals from these nuclei in order to quantify signal from individual cells. They are thus lower throughput. On the other hand, our methods permit quantification of more reporters at once because we do not have to use a fluorescence channel to track nuclei. Another important distinction is that we developed these methods with a single, different intention, to allow precise quantification of single cell reporter gene expression by diminishing sources of measurement variation. Our experiments in this study reinforce the point that cell-specific expression of reporters in multicopy arrays can differ from that of single copy genes [37], and prescribe use of single copy reporters integrated at defined sites, rather than the multicopy transgenes integrated in unknown copy number at uncontrolled/unknown loci used in the more sophisticated automated work above. Finally, it seems at least possible that the XFPs used in the work of Murray et al. might have some effect on gene expression. The GFPs used to mark nuclei are incorporated into core nucleosomes in lieu of H2B and H3.3 while the mCherry and dsRed reporters presumably exchange with native H1.1 and may participate in the same chromatin regulatory events including H1.1 mediated gene silencing. The fact that the XFP proteins that comprise our signals are not incorporated into chromatin might thus avoid yet another potential contribution to measurement variation.

### Increased reporter copy number correlates with increased reporter expression

Homozygous animals with a single copy *P*_*hsp-16*.*2*_ *-GFP* reporter on both autosomal copies of chromosome II expressed almost exactly twice as much GFP signal as F1 heterozygotes with that reporter on only one homolog (Fig 1B). That is, between one and two copies of the gene, the relationship between gene dosage and expressed gene product was linear. Such a linear relationship between gene dosage and gene product has been observed for genes on R factor and F' plasmids in *E*. *coli* [38–40], and, implicitly in *C*. *elegans*, in studies of X-linked dosage compensation, including for example, mRNA from the autosomal *myo-1* gene [41]. These results (linear relationship) are in contrast with results of two earlier studies [42,43] and also with what we observed at higher copy number here. In one prior study, strains with increased numbers of an *unc-54* transgene did not show marked increase in a quantitative measure of complementation of the *unc-54* mutant phenotype [43]. In another prior report, strains with higher copy numbers of reporter genes did not show overall monotonic increase in measured expression as copy number increased [42]. In this study, at higher copy numbers of integrated *P*_*hsp16*.*2*_ *-XFP* reporters, expression no longer increased linearly with gene dosage (e.g., 20 copies did not produce 20X more expression than one copy). We imagine that this nonlinear relationship could be due to several factors including: sequence alterations in some genes in the multicopy arrays, titration of transcription activators (below), inhibition by antisense RNA (transcribed from differently oriented genes in the multicopy arrays and/or antisense transcripts from *hsp-16*.*2*’s bidirectional promoter [44]), and silencing of repetitive DNA sequences (additional details in S1 Text, Section 4).

We observed one instance in which strains with the same number of reporter copies showed different reporter expression levels; strains CL2070 and CL2074 were both integrated somewhere in Chromosome V and had similar copy number (~20), but CL2074 was expressed 43% more (S1 Table). It is possible that the difference in expression was due to a difference in site of integration into the chromosome, that is to say, to a position effect as in [45], first discovered in [46]. However, the multicopy transgenes are different. Due to our lack of insight into the other mechanisms that might lead to differences in expression of the different transgene arrays (given above), we are reluctant to attribute the differences in expression to differences in chromosomal insertion site.

### Different reporters show identical whole-animal-to-whole-animal variation in reporter expression

All reporter strains expressed fluorescent protein only after heat shock [44,47]. All nine measured *P*_*hsp-16*.*2*_ strains showed similar animal-to-animal variation in gene expression. That is, animal-to-animal variation in reporter expression was constant even though the different *P*_*hsp-16*.*2*_ reporters had different insertion sites, fluorescent proteins, 3' UTRs/transcription terminators, and copy numbers. The fact that animals with 530 copy and single copy reporters (in TJ375 and TJ3001 animals) showed identical animal to animal expression variation is consistent with the fact that total reporter expression correlated equally with lifespan [11]. These results suggest that animal-to-animal variation in reporter expression is not affected by differences in the reporter genes themselves, but might have other sources. Our working hypothesis is that some of these differences in *C*. *elegans* may be due to differences, in physiological state variables, which we previously defined in yeast. These include persistent differences in *P*, the ability to send signals through specific pathways, and differences in *G*, the general ability to express genes into proteins [3].

### Different reporters have differently stereotyped patterns of cell-specific expression

Compared with the single copy strains, multicopy *P*_*hsp-16*.*2*_ reporter strains showed differences in cell-specific pattern of expression. For example, in TJ3001, the single copy strain, the four cells of ring I, derived from E_a_, the anterior gut lineage, showed the highest signal (see, for example, S1 Fig). By contrast, in TJ375, the 530 copy strain, the two cells of ring VIII, derived from E_p_, the posterior gut lineage, showed highest signal (see, for example, the top row of Fig 6). Two cells in the 530 copy strain showed significantly greater variation in expression level among different animals than the same cells in the single copy strain, even though the increased photomultiplier tube gain needed to image the single copy reporter likely introduced some additional noise into that measurement. Restated, the cell-specific pattern of expression from 530 copy repetitive array *P*_*hsp-16*.*2*_ *-GFP* reporter strain was distinct from the single copy reporter and at least somewhat more variable. Moreover, animals that carried two different multicopy *P*_*hsp-16*.*2*_ reporters on two different autosomes showed different and variable cell-to-cell expression (compared to animals carrying single copy reporters; Fig 6). These findings suggest that, to minimize variation in expression attributable to multicopy transgene artifacts, future studies of cell-to-cell variation should use single copy reporters.

What causes the difference in expression pattern in the multicopy *P*_*hsp-16*.*2*_ reporter strains? It is tempting to suggest that the difference in expression may be due in part to titration of limiting factors. In that strain, the combination of reporter copy number and ploidy (32X) in intestinal cells means that each intestinal nucleus contains >15,000 copies (33 nanomolar concentration) of the *P*_*hsp-16*.*2*_ promoter, which at the limit should bind >45,000 molecules of the trimeric transcription regulator HSF-1. This may be a sufficient number of gene regulatory regions and/or DNA-bound transcription regulators to titrate some protein or proteins required for *P*_*hsp-16*.*2*_ expression. Such effects on gene expression due to titration of factor(s) by transcription regulatory proteins (called "squelching"), are established in yeast [48]. It is also possible that some expression variation found in the 530 copy strain may be due to other causes, such the variable operation of the somatic systems that silence, or antagonize the silencing of, repeated DNA elements (reviewed in S1 Text, Section 4), and possible interference by antisense RNAs expressed from the bidirectional *hsp-16*.*2* promoter.

### Possible paths to future improvement of reporter expression measurement

In principle, it is possible that some quantification might be improved by genetic alterations to the animal. For example, in *C*. *elegans* it might be possible to decrease cell-to-cell variation in gene expression from reporters in multicopy arrays by altering genes involved in silencing of repeated DNA. Similarly, in intestinal cells, it is possible to diminish background cytoplasmic autofluorescence by using animals with lesions in "*glo*" genes (*glo-1*, *-2*, *-3*, and *-4*)[49], which encode proteins needed for cell functions including lysosome function, and lack gut granules [50]. Of course, any genetic "improvements" might perturb organismic function. Thus, we prefer to focus on making computational improvements, minimizing perturbations to the biological system whenever possible. It should be possible to automate delineation of the nuclear boundary, and to automate digital “stitching” of overlapping z-stacks of the same animal into a single image file. Other improvements may come from improved image acquisition, for example by using fluorescent lifetime imaging microscopy (FLIM) [51], to increase signal to noise ratio by exploiting the fact that XFP lifetimes are shorter than that of autofluorescence background [52], and perhaps to boost signal further by using specifically engineered shorter lifetime XFPs [53].

### Conclusion

We learned to definitively identify the individual cells in the adult *C*. *elegans* intestine. We devised quantification methods that minimized measurement error and used them to monitor expression of well-behaved reporter genes in all the cells of live animals’ tissue at a particular stage of adulthood. Our results establish means to identify differences in quantitative reporter expression of particular cells and groups of cells, due, for example, to differences in tissue location or developmental history [24], and should help enable genetic and molecular studies to understand the origin of these differences. These methods should also enable use of real time changes in reporter expression in cells in tissues as quantitative phenotypes in genetic screens for mutant phenotypes. The relatively short time required for data collection and the consequent diminution of cumulative measurement-induced variation should facilitate future longitudinal studies examining reporter expression from individual cells in the same animal at different points during its development and adult life. Finally, by analogy with work in *S*. *cerevisiae*, diminishing measurement error [9] may facilitate identification and study of persistent physiological states that contribute to real variation in signaling and gene expression [3]. Better understanding of these different sources of phenotypic variation will lead to a better understanding of their consequences, and might suggest ways to manipulate them.

## Supporting Information

S1 Fig The layout of the intestine cells in an adult hermaphrodite. Animal images are oriented anterior to the left and dorsal to the top. Red arrows point to anatomical features. Green arrows point to cells. **A)** Shows a cartoon diagram of an adult hermaphrodite detailing the intestine cells and relevant anatomical features. Physical orientation of all 20 intestine cells is shown. Cells are identified by proper intestine cell name, progenitor cell, and number. Green arrows point to the two occluded right cells in ring I. Alternating black and gray arrows point to black and gray lines running parallel to different intestine rings. The left handed helical half twist is pointed to by a large red arrow. Smaller red arrows show the pharynx, vulva and anus. **B)** Shows two optical slices of an adult hermaphrodite. Individual intestine rings are highlighted by alternating light gray and dark gray parallel lines. Intestine cells are labeled by their name in each optical slice. Small red arrows point to the spermathecae and the vulva. An opaque red line highlights the lumen of the intestine in the top micrograph. **C)** Shows the same two optical slices as in **B**, but with the addition of opaque purple lines delineating visually-discernible intestine cell boundaries.
(TIF)

S2 Fig Single timepoint determination of Fluorescence Not Recovered After Photobleaching (i.e., FNRAP). Fluorescence not recovered after photobleaching is shown. We measured the amount of signal recovery after photobleaching a small spot in the nucleus and the cytoplasm of 10 intestine cells in three different animals. **A)** Boxplots of the percent of original signal not yet recovered for a given area in the cytoplasm or nucleus are shown. Y axis shows the amount of signal not recovered after the photobleaching event. Top and bottom bounds of box are 25th and 75th percentile. Line in box marks median; dotted line marks mean. Top and bottom whiskers represent 10th and 90th percentiles, and “X”s denote values outside these bounds. **B)** Bar graph showing the ratio of fluorescence not recovered after photobleaching between the nucleus and cytoplasm.
(TIF)

S3 Fig Autofluorescence and GFP signals in the intestine cells of *C*. *elegans*. A) A confocal emission spectrum micrograph of *C*. *elegans* expressing the *zSi3001* single copy *P*_*hsp-16*.*2*-_ *GFP* reporter allele is shown. Sample was excited by a 488nm laser. We collected emission from 503–556 nm with 8.9 nm resolution. GFP appears as its natural teal (greenish blue) color. Autofluorescence appears as its natural greenish color. **B)** An xyz plot of the same image in **A**, but with the PMT counts for each pixel displayed in the z dimension. Z faces the reader in the top panel of **B**, and then, the top of the image is rotated away from the reader to reveal z peaks in each subsequent image below. Note that the nuclear signal peaks are smaller than the autofluorescent peaks. **C)** The red boxed area in **A** is enlarged to detail the pure GFP signal in the nucleus and autofluorescence signal in the cytoplasm in “gut granules”. **D)** Emission spectra of the two structures identified in **C** are shown. The spectrum for GFP matches published data, and is distinct from that of the autofluorescent gut granule, where emission peaks at longer wavelengths.
(TIF)

S4 Fig Attenuation of measured fluorescent protein signal with depth into intestine cell. Different cells expressing GFP or mCherry were sampled at the depths indicated in the figure legend. That is, we measured a given region of interest in the same x,y coordinates while changing optical slice depth into that cell. We then normalized values to the starting, objective- proximal sampling position, thus setting each starting value to 1 and subsequent values to some fraction of that as a function of depth. For a given sample in a given cell, the ability to measure signal loss as a function of depth in the cell was limited by the size of the individual cell, obstruction in z, and/or the size limits of the nucleus. Signal attenuated with depth at a similar rate for GFP and mCherry.
(TIF)

S5 Fig Variation in *P*_*hsp-16.2*_ *-GFP* reporter expression within experiments and between different experiments. Different dashed lines designate different populations. **A)** Variation within experiments. Smoothed histograms of *P*_*hsp-16*.*2*_ *-GFP* expression quantified in flow from three different populations of TJ375 (530 *P*_*hsp16*.*2*_ *-GFP* copies) animals heat shocked on the same day in different flasks. These runs quantified expression from 258, 435 and 632 animals. Average expression values for these populations were not significantly different (*P* > 0.6). **B)** Variation among different experiments on different days. Smoothed histograms show *P*_*hsp-16*.*2*_ *-GFP* expression quantified in flow from three different populations of TJ375 animals grown and heat shocked on three different days, sampled at 500 animals per population. Average expression values for these populations were different on different days (P < 0.05).
(TIF)

S6 Fig Demonstrative identification of the eight nuclei in the four cells comprising the left-handed helical half-twist in images from a confocal microscope. Images of RBW2 animals are shown. Top two rows show both fluorescent channels, mCherry and Emerin-GFP. Bottom row shows only Emerin-GFP. In top two rows, images show the left side of the worm and progress to the right side of the worm, starting from the top left image, ending at the bottom right image in the second row. In the bottom row, images show the left side of the worm and progress to the right side of the worm. All eight nuclei from all four cells in the intestine twist are identified in each of these two series of images; nuclei are designated by white text.
(TIF)

S7 Fig How to properly identify nuclei at the equatorial plane. Three 9-image panels displaying unannotated (left panel), annotated (middle panel), and mock-segmented (right panel) confocal images traversing through the z axis of a worm expressing GFP in its intestine cells are shown. The top left image in the middle panel denotes the three intestinal rings shown in the series of images; top is dorsal, right is anterior. For the purpose of instructing researchers to identify the equatorial plane of nuclei, the images are focused on cells in the first two rings, contained entirely in this field of view. The plane of focus moves from the right side of the animal to the left side of the animal, as images progress deeper into the worm, from the top left to the bottom right, within each panel. In order to visually detect the nuclei deeper in the intestine tissue, brightness and contrast are increased, starting in the middle row, middle column to the end of the picture series. The left panel shows a raw image of intestine cells. This is the type of image any investigator wishing to quantify reporter gene expression (in the gut cells) must face. The middle panel is annotated to show when the equatorial plane is reached when traversing through the z plane for the six nuclei in the six cells in intestine rings I and II. Yellow color overlays highlight when the equatorial plane is reached for a given nucleus along with text annotation of that nucleus. Opaque purple lines delineate visually-discernible cell boundaries. The right panel shows what it will look like when the nuclear boundary is hand drawn in ImageJ.
(TIF)

S1 Table Strains, Reporters and Expression Levels. (PDF)

S2 Table Statistics. (PDF)

S3 Table Mean and CV of Reporter Gene Expression. (PDF)

S1 Text Supporting Information. This document contains the text for S1 Text, Sections 1–7.
(DOCX)
