# Supplementary figures and images for: Single Cell Quantification of Reporter Gene Expression in Live Adult Caenorhabditis elegans Reveals Reproducible Cell-Specific Expression Patterns and Underlying Biological Variation
Source: PLoS One. 2015 May 6;10(5):e0124289. doi: 10.1371/journal.pone.0124289 (PMC4422670; doi:10.1371/journal.pone.0124289)

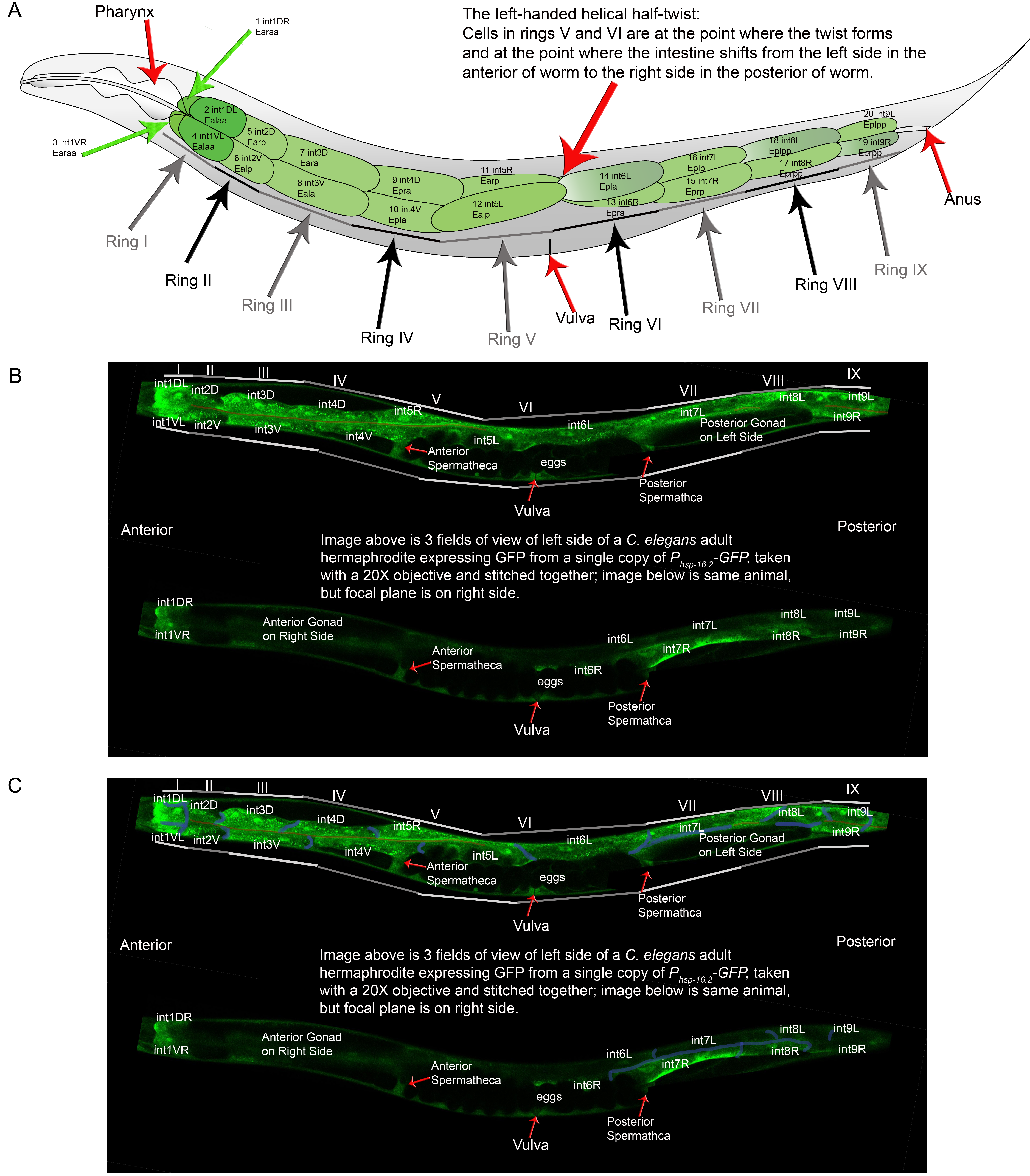

Supplement: S1 Fig — Animal images are oriented anterior to the left and dorsal to the top. Red arrows point to anatomical features. Green arrows point to cells. A) Shows a cartoon diagram of an adult hermaphrodite detailing the intestine cells and relevant anatomical features. Physical orientation of all 20 intestine cells is shown. Cells are identified by proper intestine cell name, progenitor cell, and number. Green arrows point to the two occluded right cells in ring I. Alternating black and gray arrows point to black and gray lines running parallel to different intestine rings. The left handed helical half twist is pointed to by a large red arrow. Smaller red arrows show the pharynx, vulva and anus. B) Shows two optical slices of an adult hermaphrodite. Individual intestine rings are highlighted by alternating light gray and dark gray parallel lines. Intestine cells are labeled by their name in each optical slice. Small red arrows point to the spermathecae and the vulva. An opaque red line highlights the lumen of the intestine in the top micrograph. C) Shows the same two optical slices as in B, but with the addition of opaque purple lines delineating visually-discernible intestine cell boundaries. (TIF) [file pone.0124289.s001.tif]

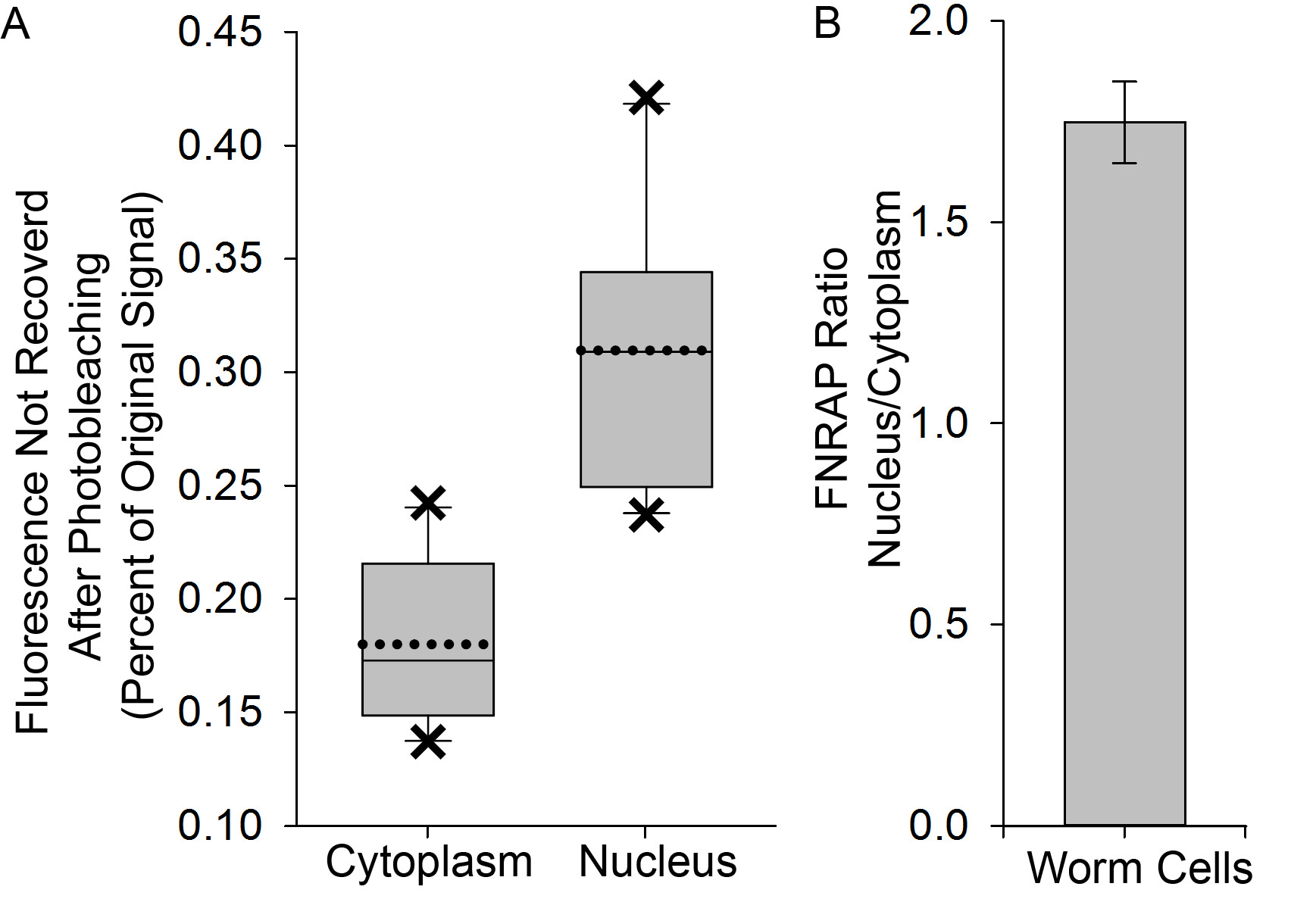

Supplement: S2 Fig — Fluorescence not recovered after photobleaching is shown. We measured the amount of signal recovery after photobleaching a small spot in the nucleus and the cytoplasm of 10 intestine cells in three different animals. A) Boxplots of the percent of original signal not yet recovered for a given area in the cytoplasm or nucleus are shown. Y axis shows the amount of signal not recovered after the photobleaching event. Top and bottom bounds of box are 25th and 75th percentile. Line in box marks median; dotted line marks mean. Top and bottom whiskers represent 10th and 90th percentiles, and “X”s denote values outside these bounds. B) Bar graph showing the ratio of fluorescence not recovered after photobleaching between the nucleus and cytoplasm. (TIF) [file pone.0124289.s002.tif]

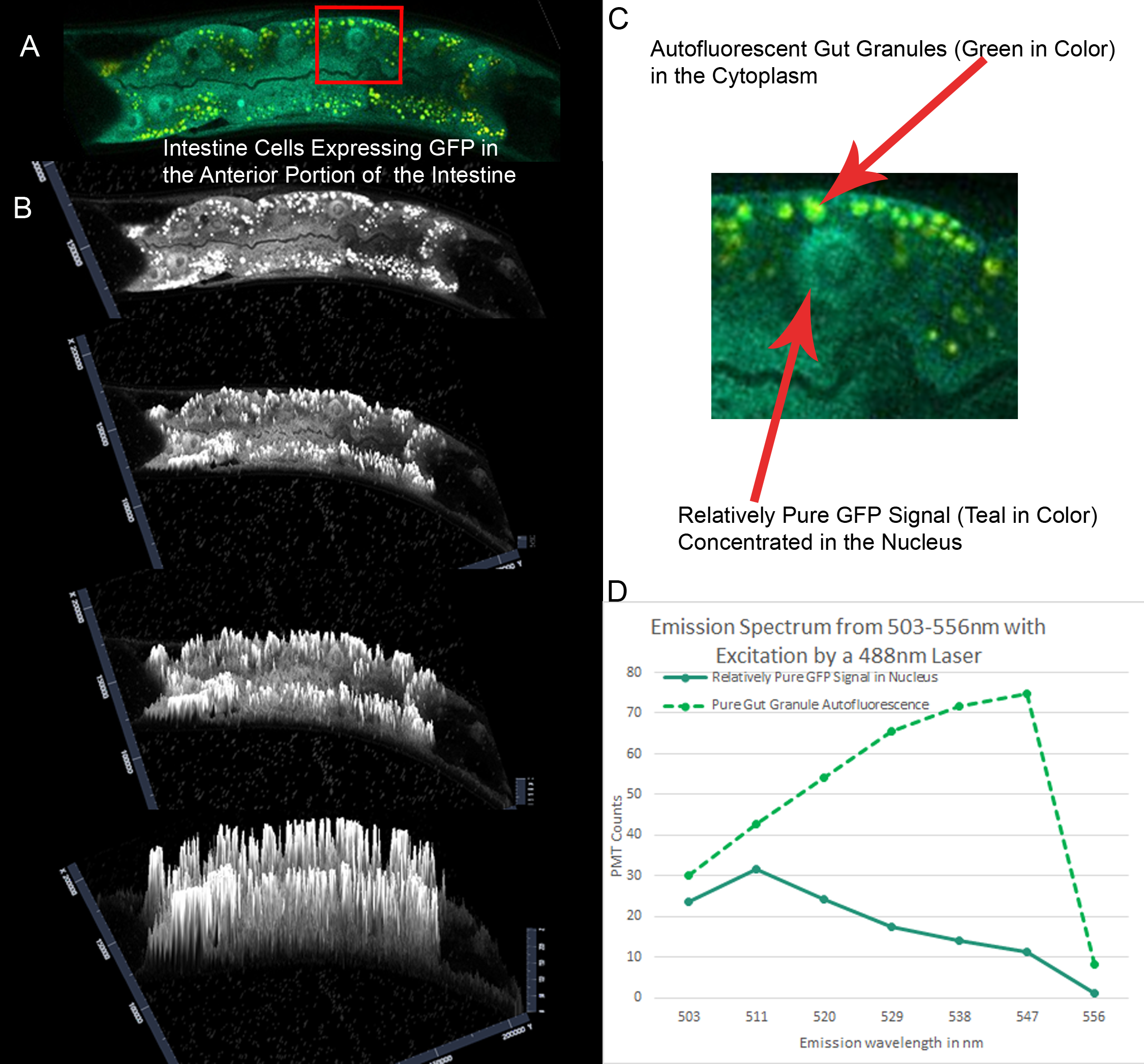

Supplement: S3 Fig — A) A confocal emission spectrum micrograph of C. elegans expressing the zSi3001 single copy P hsp-16.2- GFP reporter allele is shown. Sample was excited by a 488nm laser. We collected emission from 503–556 nm with 8.9 nm resolution. GFP appears as its natural teal (greenish blue) color. Autofluorescence appears as its natural greenish color. B) An xyz plot of the same image in A, but with the PMT counts for each pixel displayed in the z dimension. Z faces the reader in the top panel of B, and then, the top of the image is rotated away from the reader to reveal z peaks in each subsequent image below. Note that the nuclear signal peaks are smaller than the autofluorescent peaks. C) The red boxed area in A is enlarged to detail the pure GFP signal in the nucleus and autofluorescence signal in the cytoplasm in “gut granules”. D) Emission spectra of the two structures identified in C are shown. The spectrum for GFP matches published data, and is distinct from that of the autofluorescent gut granule, where emission peaks at longer wavelengths. (TIF) [file pone.0124289.s003.tif]

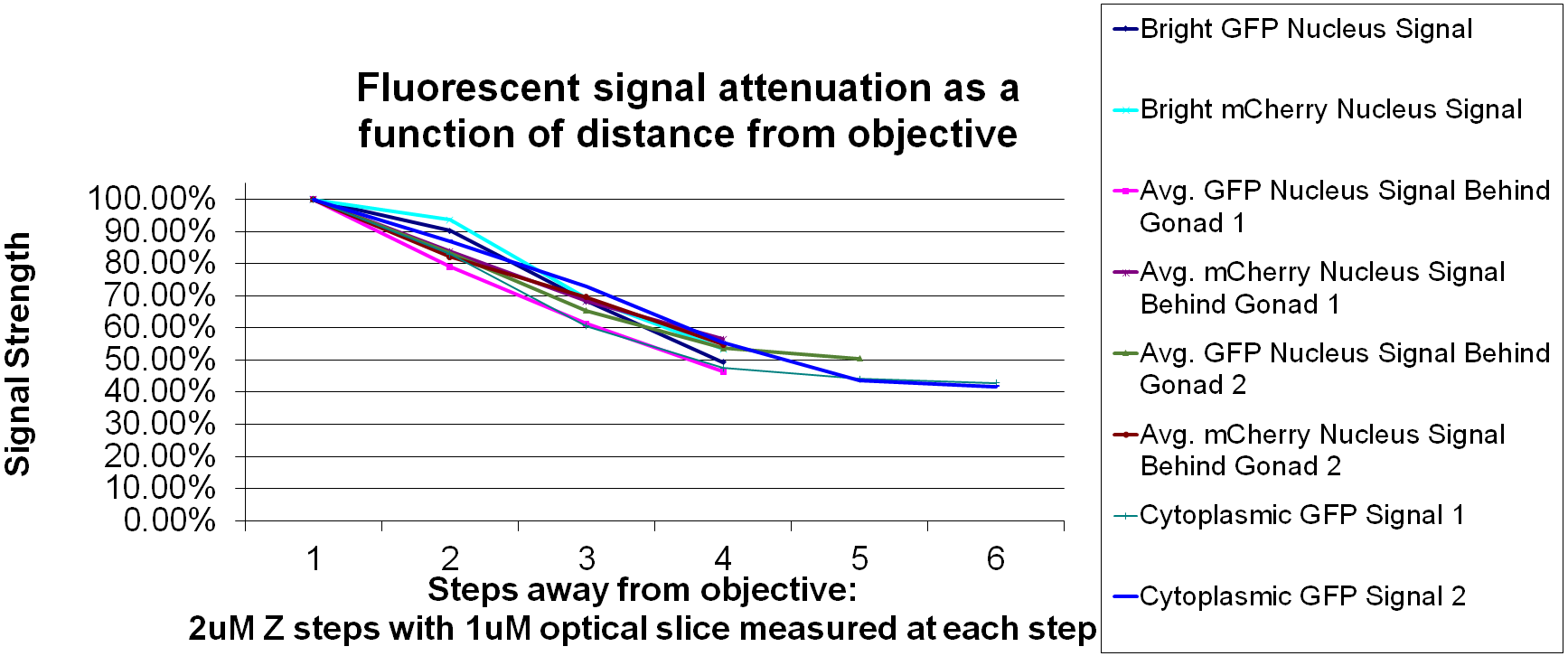

Supplement: S4 Fig — Different cells expressing GFP or mCherry were sampled at the depths indicated in the figure legend. That is, we measured a given region of interest in the same x,y coordinates while changing optical slice depth into that cell. We then normalized values to the starting, objective- proximal sampling position, thus setting each starting value to 1 and subsequent values to some fraction of that as a function of depth. For a given sample in a given cell, the ability to measure signal loss as a function of depth in the cell was limited by the size of the individual cell, obstruction in z, and/or the size limits of the nucleus. Signal attenuated with depth at a similar rate for GFP and mCherry. (TIF) [file pone.0124289.s004.tif]

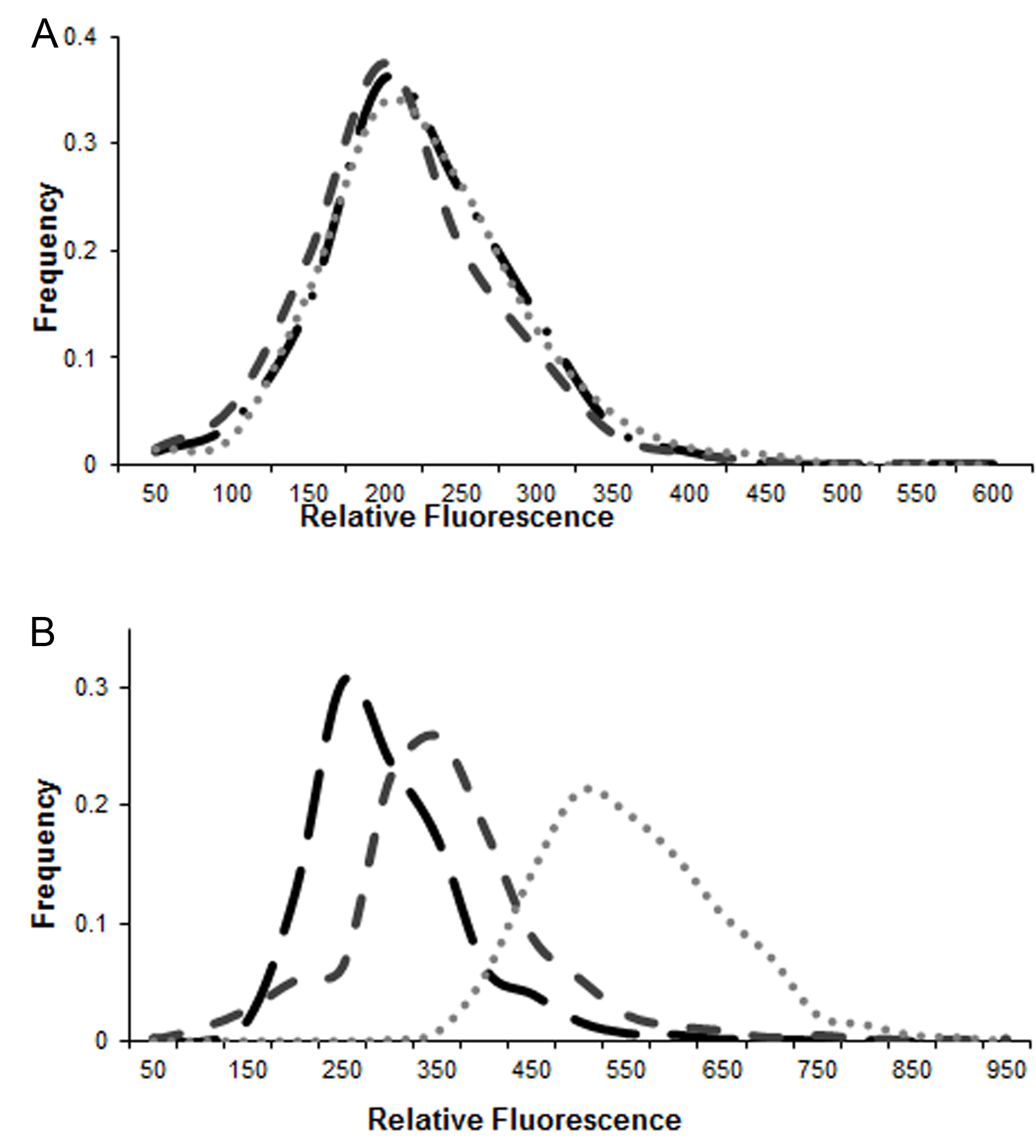

Supplement: S5 Fig — Different dashed lines designate different populations. A) Variation within experiments. Smoothed histograms of P hsp-16.2 -GFP expression quantified in flow from three different populations of TJ375 (530 P hsp16.2 -GFP copies) animals heat shocked on the same day in different flasks. These runs quantified expression from 258, 435 and 632 animals. Average expression values for these populations were not significantly different (P > 0.6). B) Variation among different experiments on different days. Smoothed histograms show P hsp-16.2 -GFP expression quantified in flow from three different populations of TJ375 animals grown and heat shocked on three different days, sampled at 500 animals per population. Average expression values for these populations were different on different days (P < 0.05). (TIF) [file pone.0124289.s005.tif]

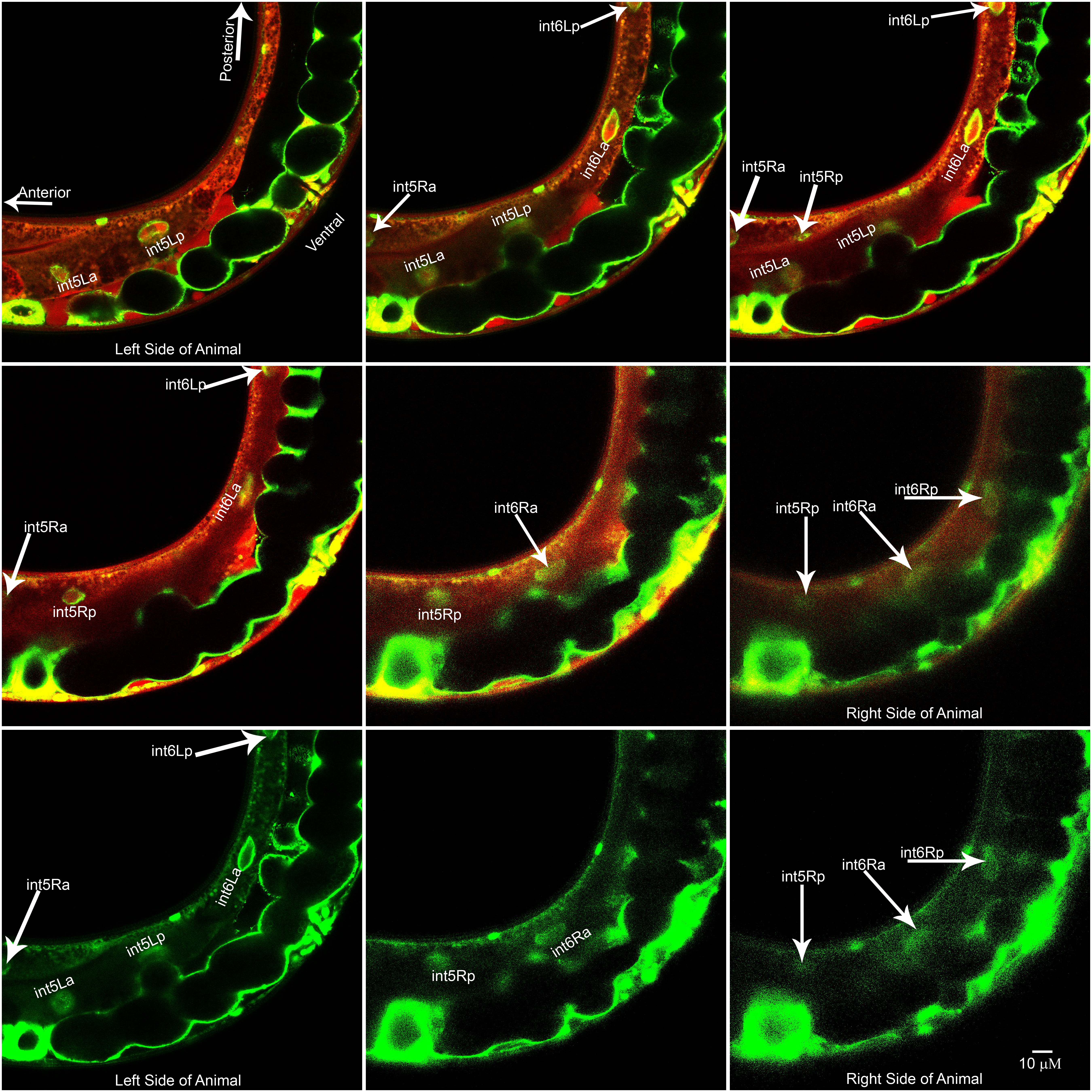

Supplement: S6 Fig — Images of RBW2 animals are shown. Top two rows show both fluorescent channels, mCherry and Emerin-GFP. Bottom row shows only Emerin-GFP. In top two rows, images show the left side of the worm and progress to the right side of the worm, starting from the top left image, ending at the bottom right image in the second row. In the bottom row, images show the left side of the worm and progress to the right side of the worm. All eight nuclei from all four cells in the intestine twist are identified in each of these two series of images; nuclei are designated by white text. (TIF) [file pone.0124289.s006.tif]

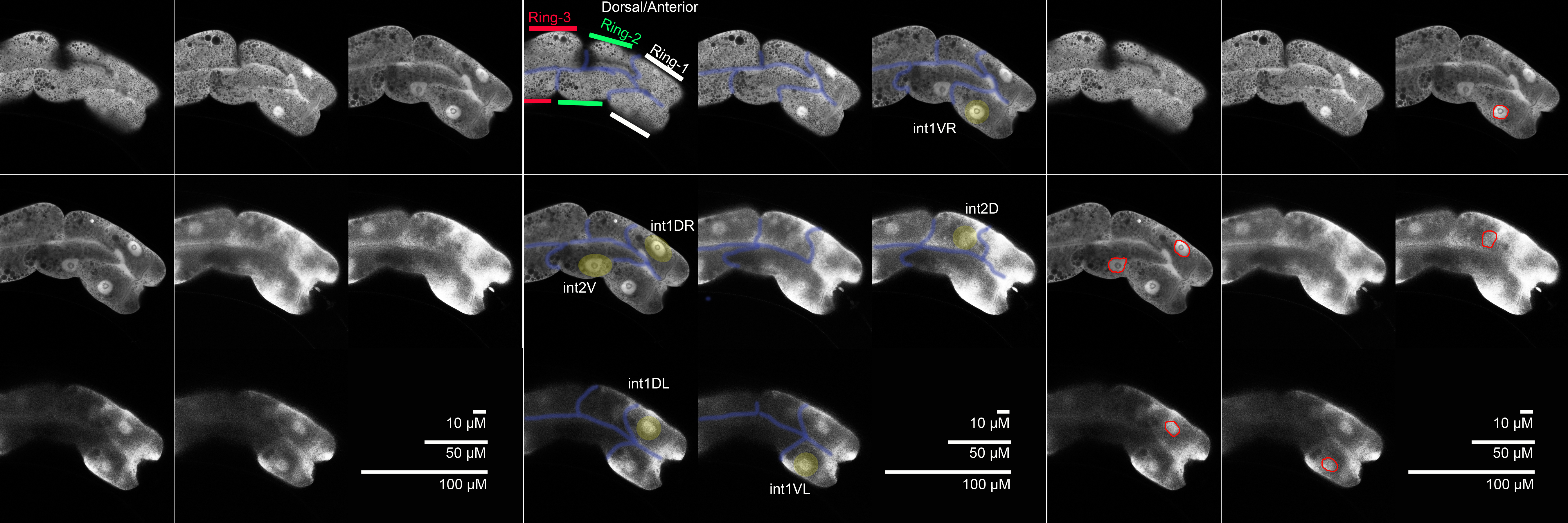

Supplement: S7 Fig — Three 9-image panels displaying unannotated (left panel), annotated (middle panel), and mock-segmented (right panel) confocal images traversing through the z axis of a worm expressing GFP in its intestine cells are shown. The top left image in the middle panel denotes the three intestinal rings shown in the series of images; top is dorsal, right is anterior. For the purpose of instructing researchers to identify the equatorial plane of nuclei, the images are focused on cells in the first two rings, contained entirely in this field of view. The plane of focus moves from the right side of the animal to the left side of the animal, as images progress deeper into the worm, from the top left to the bottom right, within each panel. In order to visually detect the nuclei deeper in the intestine tissue, brightness and contrast are increased, starting in the middle row, middle column to the end of the picture series. The left panel shows a raw image of intestine cells. This is the type of image any investigator wishing to quantify reporter gene expression (in the gut cells) must face. The middle panel is annotated to show when the equatorial plane is reached when traversing through the z plane for the six nuclei in the six cells in intestine rings I and II. Yellow color overlays highlight when the equatorial plane is reached for a given nucleus along with text annotation of that nucleus. Opaque purple lines delineate visually-discernible cell boundaries. The right panel shows what it will look like when the nuclear boundary is hand drawn in ImageJ. (TIF) [file pone.0124289.s007.tif]
