# Supplementary material for: Single Cell Quantification of Reporter Gene Expression in Live Adult Caenorhabditis elegans Reveals Reproducible Cell-Specific Expression Patterns and Underlying Biological Variation
Source: PLoS One. 2015 May 6;10(5):e0124289. doi: 10.1371/journal.pone.0124289 (PMC4422670; doi:10.1371/journal.pone.0124289)
Supplement: S1 Table — (PDF) [file pone.0124289.s008.pdf]

Strain	Copy #[a]	Mean Exp.[b]	CV (%)[c]	Chr.	Trans-gene Allele	Construct Details	Method of Integration	Transgene Configuration
TJ375	530	381	22	**IV**	*gpIs1*	$P_{hsp-16.2}$*::gfp::unc-54*	Gamma	Simple Array
TJ2735[d]	450	409	26	**V**	*zIs2735*	$P_{hsp-16.2}$*::gfp::unc-54*	UV	Simple Array
TJ2732	500	73	24	**V**	*zIs2732*	$P_{hsp-16.2}$*::dsRed::let-858*	UV	Simple Array
TJ2733	940	90	23	**IV**	*zIs2733*	$P_{hsp-16.2}$*::dsRed::let-858*	UV	Simple Array
CL2070	20	70	24	**V**	*dvIs70*	$P_{hsp-16.2}$*::gfp::unc-54; rol-6(su1006)*	Gamma	Complex Array
CL2071	22	76	26	**III**	*dvIs71*	$P_{hsp-16.2}$*::gfp::unc-54; rol-6(su1006)*	Gamma	Complex Array
CL2074	21	103	25	**V**	*dvIs74*	$P_{hsp-16.2}$*::gfp::unc-54; rol-6(su1006)*	Gamma	Complex Array
TJ3000[d]	1	29	26	**II**	*zSi3000*	$P_{hsp-16.2}$*::gfp::unc-54; cb-unc-119(+)*	MosSCI	Single Copy
TJ3001	1	32	23	**II**	*zSi3001*	$P_{hsp-16.2}$*::gfp::unc-54; cb-unc-119(+)*	MosSCI	Single Copy

a - Copy number was determined via qPCR, using *ama-1* as a control.

b – Mean expression level for each reporter derived from several measurements of population mean. Each value was the measured average photomultiplier tube counts.

c - No CV for any *hsp-16.2* reporter was found to be significantly different from any other *hsp-16.2* reporter.

d - These strains were measured thrice; they were not measured as many times as the other reporters. All other strains were measured in together in a series of five or ten experiments (See Materials and Methods). Data shown for TJ375 is from the five experiments with other multicopy reporters in experimental series one. Mean from the ten experiments with TJ3001 is shown in Table 2. Mean expression values for the single copy reporters are not significantly different from one another.
