# Supplementary material for: Single Cell Quantification of Reporter Gene Expression in Live Adult Caenorhabditis elegans Reveals Reproducible Cell-Specific Expression Patterns and Underlying Biological Variation
Source: PLoS One. 2015 May 6;10(5):e0124289. doi: 10.1371/journal.pone.0124289 (PMC4422670; doi:10.1371/journal.pone.0124289)
Supplement: S2 Table — (PDF) [file pone.0124289.s009.pdf]

Parameter Analyzed	Data Source	Statistical Test	Factor A	Factor B	Factor Analyzed	F (t)	P
CV	Series One Flow	One Way Repeated Measures ANOVA	Strain	NA	Strain	0.98	0.45
CV	Series One Flow	Two Way ANOVA	Fluor	Chromosome	Fluor	1.87	0.18
CV	Series One Flow	Two Way ANOVA	Fluor	Chromosome	Chromosome	0.74	0.49
CV	Series One Flow	Two Way ANOVA	3'UTR	Array Type	3'UTR	2.93	0.10
CV	Series One Flow	Two Way ANOVA	3'UTR	Array Type	Array Type	1.20	0.28
CV	Series One Flow	Two Way ANOVA	Array Type	Fluor	Array Type	1.20	0.28
CV	Series One Flow	Two Way ANOVA	Array Type	Fluor	Fluor	2.93	0.10
CV	Series Two Flow	Paired t-test	Strain	NA	Strain	NA	0.23
Mean	Series Two Flow	Paired t-test	Strain	NA	Strain	NA	0.00
Mean	CL2070 and CL2074 Flow Data	Paired t-test	Strain	NA	Strain	NA	0.04
CV	Image Cytometry Cell Positions	Two Way Repeated Measures ANOVA	Strain	Cell	Strain in int5R	(2.71)	0.01
CV	Image Cytometry Cell Positions	Two Way Repeated Measures ANOVA	Strain	Cell	Strain in int6L	(2.40)	0.02
CV	Image Cytometry Summed Cells	Paired t-test	Strain	NA	Strain	NA	0.41
