# Supplementary material for: Single Cell Quantification of Reporter Gene Expression in Live Adult Caenorhabditis elegans Reveals Reproducible Cell-Specific Expression Patterns and Underlying Biological Variation
Source: PLoS One. 2015 May 6;10(5):e0124289. doi: 10.1371/journal.pone.0124289 (PMC4422670; doi:10.1371/journal.pone.0124289)
Supplement: S3 Table — (PDF) [file pone.0124289.s010.pdf]

Strain	Standard	Standard, One Week Starved L1	Ultrasynchronized, No Starved L1	Ultrasynchronized, hatchout at 0.1 animal/µL	Ultrasynchronized, hatchout at 1 animal/µL
TJ375	265, 18	180, 21	290, 19	238, 21	Dropped Sample
TJ3001	39, 17	34, 25	32, 18	29, 19	29, 17

Mean and CV determined from measurement of approximately 500 animals in flow. Data is presented as: Mean (arbitrary PMT counts), CV (as a percentage).
