# Supplementary material for: Single Cell Quantification of Reporter Gene Expression in Live Adult Caenorhabditis elegans Reveals Reproducible Cell-Specific Expression Patterns and Underlying Biological Variation
Source: PLoS One. 2015 May 6;10(5):e0124289. doi: 10.1371/journal.pone.0124289 (PMC4422670; doi:10.1371/journal.pone.0124289)
Supplement: S1 Text — This document contains the text for S1 Text, Sections 1–7. (DOCX) [file pone.0124289.s011.docx]

**S1 TEXT: SUPPORTING INFORMATION**

**Section 1) The adult *C. elegans* intestine**

In adult hermaphrodite animals, the intestine cells form a long (more than ¾ body length) tube surrounding the intestinal lumen. The 20 cells are organized into nine roughly cylindrical segments called rings, starting from ring I near the pharynx, running posteriorly through ring IX. Ring I is comprised of four cells, with each cell forming a quarter cylinder. Each of the subsequent rings is comprised of two cells with each cell forming a half cylinder (Figures 2 and S1).

The cells are relatively large, from 35-150 microns in length, and contain correspondingly large (10 x 10 x 12 microns) ellipsoidal nuclei. Like some cells in vertebrate tissues including cardiomyocytes [[1](#_ENREF_1)] and hepatocytes [[2](#_ENREF_2)], the cells are polyploid (32n or 16 diploid genome copies per nucleus) and sometimes binucleate [[3](#_ENREF_3),[4](#_ENREF_4)]. The increased ploidy is generally thought to facilitate increased production of mRNAs and thus enhanced metabolic capacity [[5](#_ENREF_5)]. In fact, this tissue breaks down enough food to enable hermaphrodites to produce up to their own mass in eggs every 24 hours [[6](#_ENREF_6)].

The 20 cells arise from a single cell in the 8-cell embryo, the “E” (for endoderm) cell [[3](#_ENREF_3),[7-9](#_ENREF_7)]. This E lineage is practically invariant, and the way the cells divide and form the tissue is stereotyped. During embryogenesis, the descendants of E form two rows of eight cells, one on the left and one on the right, and the anteriormost and posteriormost cells divide once more [[7](#_ENREF_7),[8](#_ENREF_8)]. By the time an animal hatches, all twenty intestine cells are present and spatially oriented [[3](#_ENREF_3)] as they are in the adult (see paragraph below).

In the adult, as in larvae [[3](#_ENREF_3)], the intestine has a left-handed helical half-twist (Figures 2, S1 and S6). Specifically, approaching the left-handed helical half-twist from the anterior of the animal, the ventral cell int4V connects to ventral/lateral cell int5L, which connects to lateral/dorsal cell int6L which connects to dorsal cell int7L. The consequence is to orient the Epl lineage of cells, in rings VII-IX, dorsally in the posterior of the adult animal (Figures 2 and S1; Personal Communication from Jim Priess). Due to this left-handed helical half-twist, the dorsal row of cells in the posterior of adult animals are in fact the L-lineage of cells, the same L-lineage cells that are ventral (and so named) in the anterior of the animal in rings II-IV. We provide additional details regarding the identity and means to identify each cell in Results and S1 Text, Sections 5 and 7.

To our knowledge, there had been no previous published description of the identification of adult intestine cells. Previous reports have identified embryonic intestine cells [[7-9](#_ENREF_7)]. Other prior reports, such as [[3](#_ENREF_3)], which looked at newly hatched worms, and [[4](#_ENREF_4)], which looked at adult worms, identified intestine cell nuclei but did not identify intestine cells by their names or lineal relationships.

**Section 2) Reporter genes and measurement of reporter gene expression**

Since the first *Φ 80trp-lac* fusions [[10](#_ENREF_10)], scientists have studied expression of genes that fuse heterologous promoters to the coding sequences of other genes whose products are easily quantified. Researchers have used expression from such reporters in qualitative genetic experiments to understand how initiation of mRNA transcription from the fused promoter is regulated. They have also used reporter expression to quantify and compare, using a common readout, the amounts of protein (and, by inference, mRNA) synthesized under the control of different promoters. Use of such reporters has contributed greatly to the understanding of gene regulation in essentially all organisms under current study, including the model organisms *Saccharomyces cerevisiae*, *Caenorhabditis elegans, Drosophila melanogaster*, and *Mus musculus*. For many decades, the most commonly used reporter gene products were derivatives of *E. coli* beta-galactosidase (*lacZ* product) quantified by colorimetric assays with artificial substrates, galactoside derivatives developed for bacterial genetics [[11](#_ENREF_11),[12](#_ENREF_12)]. Since the 1990s, scientists have also used fluorescent proteins, often derivatives of the Green Fluorescent Protein (GFP) from *A. victoria*, quantified by exciting samples with light of shorter wavelength, and measuring the amount of longer wavelength light the excited proteins emit [[13](#_ENREF_13)]. Use of GFP and related fluorescent proteins (here called XFPs) as readouts for gene expression in single cells has since become widespread.

Any research that attempts to draw conclusions from small quantitative differences in reporter gene expression benefits from attention to precise measurements. Such attention can be rewarding; for example, it allowed us to develop simple and surprisingly accurate methods to use expression of calibrated *lacZ* reporters to measure Kds of protein-protein interactions in the interaction trap two-hybrid system [[14](#_ENREF_14)]. Later, in *E. coli* and yeast, we and others developed accurate image-based means to quantify expression of reporter genes that express fluorescent proteins. Accurate quantification of XFP fluorescence in *Saccharomyces cerevisiae* required careful attention to issues including but not limited to: the total number of photons emitted per XFP molecule prior to photobleaching, automated means to capture a microscope image, automated identification of cells within the image, and measurement of different cellular variables (Cell-ID), as well as attention to the amount of XFP signal above autofluorescence background, the rate of XFP photobleaching, the rate of autofluorescence photobleaching, the contribution to signal from photons emitted from pixels that neighbor cells, the contribution from cell and non-cell volumes out of the focal plane, and the loss of photons to pixels outside of the assayed pixels [[15-17](#_ENREF_15)].

The ability to eliminate variation in quantified expression introduced by measurement in turn enabled experiments to study and understand true biological variation in reporter expression. In yeast, some cell-to-cell differences in expression of a reporter gene induced by the pheromone response signaling system are due to cell cycle position, and some due to stochastic noise in gene expression (first described in *E. coli* [[18](#_ENREF_18)]). However, the bulk of the non-cell-cycle variation in expression of a pheromone inducible reporter gene is due to persistent, non-genetic differences in ability of individual cells to send signals through the pathway, and to persistent cell-to-cell differences in general ability to express genes into proteins [[15](#_ENREF_15)].

**Section 3) Transgenesis and reporter genes in *C. elegans***

Methods for constructing organisms bearing reporter genes vary from organism to organism. *C*. *elegans* lacks circular plasmid or linear artificial chromosome cloning vectors. Rather, and unique to *C. elegans*, the most common approach to constructing animals with transgenic reporters has been to inject DNA containing the reporter construct into the syncytial part of the gonad, and to then select progeny that contain the transgene from eggs laid by the injected worm [[19-21](#_ENREF_19)]. These transgenic progeny contain large (usually circa 100 copy) concatamers of the injected DNA. These concatamers are usually not integrated into the worm genome (i.e., are extrachromosomal) and are referred to as extrachromosomal arrays. Upon injection, circular DNA is formed into head-to-tail concatamers (direct repeats); some of the repeats show duplications and deletions of the injected DNA [[19](#_ENREF_19)] probably at the site at which the sequences join [[20](#_ENREF_20),[22](#_ENREF_22)]. By contrast, upon injection, linear DNA is formed into concatamers with head-to-tail, head-to-head, and tail-to-tail junctions (i.e., both direct and inverted repeats), and these frequently bear deletions of sequences at the ends of the injected fragment [[19](#_ENREF_19)]. *C. elegans* chromosomes are "holocentric", in that each chromosome carries along its length numerous centromeres, situated in sites for nonspecific binding of multiple transcription factors (transcription factor hotspots; HOT sites) and defined by single cenH3-containing nucleosomes [[23](#_ENREF_23)]. Extrachromosomal arrays are often partitioned into daughter cells with significant rates of nondisjunction, making them useful for mosaic analysis [[19](#_ENREF_19),[24](#_ENREF_24)].

*C. elegans* researchers have also constructed transgenic animals in which the reporters, instead of being extrachromosomal, are integrated into the chromosome. For example, direct selection for suppression of an otherwise sterile *tra-3* nonsense mutation by a *sup-7* (Trp) amber suppressor [[25](#_ENREF_25)], which was part of a *P_hsp-70(Drosophila)_-lacZ* reporter construct injected into worm oocyte nuclei, produced strains with integrated DNA at defined chromosomal sites [[21](#_ENREF_21)]. Integrated transgenic DNA selected using *sup-7/tra-3* is often concatamerized at low copy number, possibly because too much expression of the *sup-7* marker is deleterious [[21](#_ENREF_21),[22](#_ENREF_22)]. In later work, researchers recovered constructions integrated into chromosomal DNA after injecting them into the gonadal syncytium and irradiating the animals with gamma rays [[24](#_ENREF_24)], which cause double strand breaks, or in one instance, by injecting the plasmid construct together with one of two different sequences of 50-mer single stranded oligonucleotide [[20](#_ENREF_20)]. For unknown reasons, coinjection with these short sequences favors construct integration at low copy number or even single copy without the need for irradiation [[20](#_ENREF_20)]. However, most reporters in use today have been created by injecting circular DNA, producing “repetitive” arrays, which were then either left as heritable extrachromosomal arrays or integrated into the genome.

In the late 1990s and early 2000s, because transgenes in “repetitive” arrays were silenced in the germline (see silencing in S1 Text, Section 4), researchers attempted to reduce the "repetitiveness" of transgenes. First, investigators attempted to reduce repetitiveness by diluting the plasmid containing the transgene of interest with genomic DNA before injection. Transgenes produced by co-injection of the plasmid of interest with PvuII-cut genomic DNA (whether extrachromosomal or integrated) are comprised of DNA from both sources and are said to reside in "complex" arrays [[26](#_ENREF_26)]. Later, researchers were able to make less repetitive reporter genes by introducing, into cells of the germline, transgenes on microparticles coated with plasmid DNA [[27](#_ENREF_27)], using an instrument originally called a gene gun [[28](#_ENREF_28),[29](#_ENREF_29)]. Transgene introduction by "microparticle bombardment" results (via unknown mechanisms) in strains typically bearing relatively few (1-10) copies of the plasmid sequence integrated into the genome at a random single sites [[27](#_ENREF_27)]. Inspection of Figure 2 in Praitis et al. 2001 suggests that the DNA in such transgenes is a linear head-to-tail concatamer of the incoming plasmid, and that the repeating units have sometimes suffered insertions or deletions at the points where the plasmids joined [[27](#_ENREF_27)]. However, neither method (complex array formation or bombardment) controls site of integration or copy number.

Until recently, methods to construct worm strains that carried single copies of transgenes were not well advanced. More than 20 years ago, researchers generated a strain bearing a single copy of a transgene integrated by repair of a Tc1 (Mariner family) transposon insertion with injected template DNA [[30](#_ENREF_30)]. In another instance, investigators demonstrated homologous recombination into genomic DNA with injected DNA without having taken steps to create DNA breaks [[31](#_ENREF_31)]. Later, in the early 2000s, one group reported a targeted homologous recombination into the genome after transformation by particle bombardment [[32](#_ENREF_32)]. More recently, researchers systematized the use of transposase-induced excision of another Mariner family transposon (Mos) from defined chromosomal loci to create double strand breaks, which were then repaired by injected template DNA bearing homology at its ends to the sequences flanking the chromosomal breakpoint [[33-35](#_ENREF_33)]. Still more recently, investigators have used the RNA-guided bacterial endonuclease, Cas9 [[36](#_ENREF_36)] to cut the worm genome at defined sites. The break is repaired with injected transgene DNA as above. Such repair can, if the investigator desires, result in transgenic animals in which the coding sequence of the native copy of the gene is fused to the coding sequence of an XFP [[37](#_ENREF_37)], reviewed in [[38](#_ENREF_38)].

**Section 4) Silencing of repetitive gene arrays in the *C. elegans* germline and soma**

In somatic cells, in contrast to the complete silencing of repetitive DNA sequences in germline cells (e.g., those cells descended from Z2 and Z3) [[26](#_ENREF_26),[39](#_ENREF_39)], transgenes resident in multicopy arrays are usually expressed at some level [[24](#_ENREF_24)]. However, there are cases in which expression from repetitive arrays is silenced in somatic cells [[40](#_ENREF_40),[41](#_ENREF_41)]. Furthermore, somatic expression patterns of integrated repetitive arrays are sometimes mosaic within and between animals [[24](#_ENREF_24),[40](#_ENREF_40),[42](#_ENREF_42)].

The mechanism(s) governing somatic silencing of transgene arrays are not well understood. Like heterochromatin silencing in *Drosophila* [[43](#_ENREF_43)] and in mammals [[44](#_ENREF_44)], somatic silencing of repetitive transgene arrays in *C. elegans* requires components of the RNA interference system [[45](#_ENREF_45),[46](#_ENREF_46)] including *dcr-1* (dicer) and *rde-1* (argonaute) [[47-49](#_ENREF_47)]. As in mammals [[50](#_ENREF_50)] and *Drosophila* [[51](#_ENREF_51)], somatic silencing in *C. elegans* requires a heterochromatin protein 1 (HP1) homolog, *hpl-2*, as well as a SETDB1 homolog, *met-2*, and a functional equivalent of SUV39H1/2, *set-25* [[52](#_ENREF_52)] (we note that the biochemical functions of these proteins in worms may not be completely identical). In *C. elegans*, mutation of genes involved in RNA editing (*adr-1* and *adr-2*) [[45](#_ENREF_45)], and other genes involved in chromatin modification (*tam-1* and *lex-1*) [[53-55](#_ENREF_53)] results in somatic silencing of transgenes, suggesting that these genes might normally antagonize silencing of genes in arrays. Studies using RNAi have identified additional candidate genes that might potentiate somatic silencing [[56](#_ENREF_56)]. These two additional tiers of regulation (silencing and antagonism of silencing) imposed on expression of repeated genes may further contribute to variation in somatic expression of transgenes in integrated multicopy arrays.

**Section 5) Expanded Materials and Methods**

**Strains:** Table 1 shows strains used in these experiments. We described construction of CL2070 (20 copies, chromosome V), CL2071 (22 copies, chromosome III) and CL2074 (21 copies, chromosome V) in [[57](#_ENREF_57)], construction of TJ375 (530 copies, chromosome II) in [[58](#_ENREF_58)], and construction of TJ3000 and TJ3001 (both 1 copy, chromosome II) in [[59](#_ENREF_59)]. We cultured these strains as described below.

**Strain Construction:** All eGFP (S65T and I167T) reporter constructions carried the 5’UTR (the upstream regulatory sequences including the promoter) of the *hsp-16.2* gene (i.e., *P_hsp-16.2_)* fused to an intron-free eGFP coding sequence [[57](#_ENREF_57)] and the 3’UTR of *unc-54*. In these constructions, the reason we used eGFP, and not the more usual S65C “worm GFP”, is that we used this eGFP in the strains in our original studies that found a correlation between *P_hsp-16.2_* -GFP and lifespan [[58](#_ENREF_58)]. To make the multicopy *P_hsp-16.2_-GFP* reporter strains TJ2732 and TJ2733 we used a plasmid, pPT1000. We created this plasmid by cleaving pCL148 with AgeI and EcoRI, gel-extracting the resulting 5’AgeI-dsRedMonomer-3’EcoRI DNA fragment, and ligating that into an AgeI- and EcoRI-cut, gel-extracted pPD118.26 plasmid "backbone" to replace the now absent coding sequence of GFP. The resulting plasmid, pPT1000, fused the *P_hsp-16.2_* sequence to the coding sequence for dsRed monomer [[60](#_ENREF_60)] and the *let-858* 3’UTR sequences. To make extrachromosomal array strains TJ2730 and TJ2731, we injected this plasmid at 50ng/µL into 30-40 wild-type animals until we saw the gonad expansion from the injected DNA solution as described in [[20](#_ENREF_20)]. To make TJ2735 we first injected pCL25 [[58](#_ENREF_58)] into wild-type animals at approximately 50ng/µL to generate the extrachromosomal array strain CL1082 in the same fashion we made TJ2730 and TJ2731. We then used ultraviolet radiation to induce chromosomal integration of the extrachromosomal reporters in TJ2730, TJ2731, and CL1082 as described [[24](#_ENREF_24)]. We made the single copy TJ3002 strain by cutting pLen1.8 with BamHI and EcoRV and gel-extracting the approximately 900bp mCherry fragment and ligating that into the BamHI and EcoRV cut pCL25 backbone fragment lacking eGFP to make pAM103, wherein eGFP had been swapped for mCherry. We then used pAM103 to make a MosSCI chromosome II targeting vector, pAM104, which we then integrated and validated locus and sequence as described in [[59](#_ENREF_59)]. RBW2661 and RBW2642 contained single copy *P_daf-21_-GFP* and *P_daf-21_-mCherry* reporters on Chromosome II, respectively. We made the DNA constructs encoding the *P_daf-21_-XFP* reporters by using yeast gap repair [[61](#_ENREF_61)]*.* We introduced this *P_daf-21_* reporter sequence into a vector that targeted the DNA to be inserted at the chromosome II MosSCI transposon at site ttTi5605. We then injected the construct at 50ng/µL into EG6699 animals and recovered single copy insertions that we size and site validated by PCR. We outcrossed all strains with N2CGCb at least five times, except the *P_daf-21_* strains, which were outcrossed only once. We disposed of the extrachromosomal array strains after we isolated the integrated lines. We made RBW2 worms by crossing TJ3002 worms with LW0699 worms (this strain, a gift from Kelly Liu, carried an EMR-1::GFP fusion protein gene [[62](#_ENREF_62)]), and isolating F2 animals homozygous for both reporters. We verified homozygosity by standard transmission (Mendelian) genetic analysis of the progeny of isolated F2 hermaphrodites, as previously described [[63](#_ENREF_63)]. Specifically, we isolated 32 F2 progeny from each of four F1 hermaphrodites resulting from the TJ3002 x LW06699 cross. We identified one animal that transmitted both transgenes to 100% of its progeny in each of the four groups of 32 F2 progeny; that is, we identified F2 animals that had F3 progeny that were 100% GFP and mCherry positive. We thus obtained animals we named RBW1, RBW2, RBW3 and RBW4. We arbitrarily chose RBW2 to use in this study; all four strains appeared superficially wildtype and had seemingly identical reporter expression. We maintained and cultured all strains at 20° on solid NGM plates as described in Strain Cultivation below. To make F1 animals heterozygous for zSi3001, we mated TJ3001 males with TJ3002 hermaphrodites, picked F1 hermaphrodites away from males and onto new seeded NGM plates as L4s, and subsequently analyzed the hermaphrodites in flow. As a control, we mated TJ3001 males with TJ3001 hermaphrodites, picked F1 hermaphrodites away from males as L4s, and subsequently analyzed those hermaphrodites in flow as well.

**Strain Characterization:** We described linkage group and copy number determination for TJ375, TJ3000 and TJ3001 previously [[59](#_ENREF_59)]. For CL2070, CL2071 and CL2074, TJ2732, TJ2733, and TJ2735 we mapped transgenes to individual chromosomes by performing crosses with animals carrying different scorable mutations (e.g., uncoordinated, *unc*, and dumpy, *dpy*) on each of the six chromosomes as described in [[63](#_ENREF_63)].

We inspected microscopic images of animals for all *P_hsp-16.2_-XFP* reporter strains in Table 1 cultured at 20° in the absence of heat shock. None showed visible fluorescent signal. For TJ375, we previously confirmed lack of expression with an anti-GFP antibody in Western blots [[58](#_ENREF_58)]. These results were consistent with the lack of expression of both HSP-16.2 and GFP determined by antibody staining and fluorescence microscopy, respectively, in CL2070 animals, previously described in [[57](#_ENREF_57)]. The lack of expression of *hsp-16.2* reporters in the absence of heat shock was also consistent with the prior analyses of lacZ-based reporters (microscopically) [[64](#_ENREF_64)] and the endogenous *hsp-16* genes (Northern blots) [[65](#_ENREF_65)].

We determined copy number of multicopy transgenes using Syber-green based qPCR, as described in [[66](#_ENREF_66)] and as we did previously in [[59](#_ENREF_59)]. We designed primers for GFP (F - CCTGTCCACACAATCTGCCC; R -TGGTCTCTCTTTTCGTTGGGAT), dsRed (F - TCGCCACCATGGACAACA; R - CCCTCCATGCGCACCTT), and ama-1 (F – GCGGTCAGAAAGGCTATCGA; R - AGCAGTGCCAAATGTCGGTAAT), a control present in one copy per haploid genome. All of the primers had a similar T_m_ around 60° as in [[67](#_ENREF_67)], and were designed to generate a 50-90 bp product. We extracted DNA from animals using standard phenol-chloroform extraction of starved plates of worms that had no visible *E. coli* left. We performed 25 µL PCR reactions using 200nM primer concentration for 40 PCR cycles. We performed each reaction measuring the DNA from each strain in duplicate at three different DNA concentrations (e.g., 1, 10, 100 ng of template) in three different experiments.

We used the comparative CT method to calculate copy number from these values for each of the multicopy strains [[68](#_ENREF_68)]. Briefly, we calculated copy number from each PCR reaction by measuring the fluorescence signal (proportional to the amount of dsDNA produced, which is proportional to the amount of template present) and comparing that to the fluorescence signal from the *ama-1* reaction from the same template DNA. We averaged duplicates from each experiment. We calculated values from each of the three concentrations by comparing the samples to the DNA template concentration-matched control. We averaged the copy numbers calculated from each concentration to determine the copy number for that particular strain in that particular experiment. We repeated the above three times (in three separate runs of experiments) to generate the copy number presented in Table 1. These methods have a reported accuracy of between 6-21% [[66](#_ENREF_66)]. Accordingly, the observed differences in copy number among strains CL2070 (20 copies), CL2071 (22 copies), and CL2074 (21 copies) are not significant.

**Cultivation of *P_hsp-16.2_-XFP* reporter strains:** We grew animals (for stocks and for experiments) at 20° on solid NGM media seeded with *E. coli* OP50 culture that had been grown to saturation overnight in LB media. To “seed” plates, we placed 1 mL of overnight culture on each 10 cm NGM plate, forming a bacterial lawn after drying. We synchronized populations of animals by using alkaline sodium hypochlorite solution to kill and dissolve everything but the eggs from large, well-fed, mixed-stage populations [[69](#_ENREF_69)]. We allowed surviving eggs to hatch by placing them in approximately 10mL sterile S-basal on a sterile 10cm NGM petri dish at 0.5-3 eggs per microliter at 30 RPM at 20°. After about 16 hours in the aforementioned hatching solution, this procedure resulted in L1 larvae, which had arrested in L1 because food deprivation had caused them to enter the insulin dependent L1 diapause [[70-72](#_ENREF_70)]. We settled upon this synchronization procedure after having tested others in pilot experiments, described in the next section (S1 Text, Section 6).

We initiated growth and development of synchronized starved cultures by placing animals onto fresh 10cm solid NGM Petri dishes seeded with 1mL OP50. To place the same number of animals on each Petri dish, we spun animals from the overnight hatchout solution down in a 15 mL conical centrifuge tube and resuspended them in a few milliliters of sterile M9. We placed between 5 and 100 uL of this suspension on each plate, fixing the density of animals to 175 animals per 10cm NGM Petri dish. We incubated the plates at 20° for 72 hours and then heat shocked each set.

**Heat shock Conditions:** To heat shock each population, we first washed animals from growth plates into 50-mL conical tubes using S-basal medium. We centrifuged the animals and removed the supernatant. We resuspended the animals in 50 mL of S-basal that had been pre-warmed to 35° and seeded with OP50. For experiments quantifying *P_hsp-16.2_-XFP* expression in flow, we transferred the heated worms into flasks and placed the animals in a 35° incubator for one hour, swirling around at 100 RPM, also described in [[58](#_ENREF_58)]. We let animals recover in 50 mL S-basal seeded with 1mL of saturated OP50 culture for 24 hours at 20° at 100 RPM. For experiments quantifying *P_hsp-16.2_-XFP* expression microscopically, we carried out heat shock and recovery on 6cm Petri dishes containing approximately 10 mL of NGM seeded with approximately 300 microliters of OP50.

**Quantification of whole animal fluorescence signal:** We used a COPAS Biosort 250 to measure *P_hsp-16.2_-GFP* expression in flow as in [[59](#_ENREF_59)]. This instrument has a sample cup, into which we placed the animals suspended in S-basal medium, typically with about 1000 worms in 10 mL. Once in the sample cup, the worms were whisked toward an inlet by stir bars and the suction of the inlet. The worms entered the opening mostly one at a time, and then they flowed through tubes where they were further separated and straightened by the sheath fluid stream before measurement. After hydrodynamic straightening, the machine measured, with a diode laser, both beam break time (length) and beam absorption (extinction coefficient). The machine also measured the fluorescence of each worm by exciting it with a 488nm laser and collecting the emitted green or red light with either a 510/23 or a 610/20 emission filter and then measuring that emitted green or red light with a photomultiplier tube (PMT). This entire event sequence occurred in a fraction of a second. We used these parameters (length and extinction coefficient) to “gate” the measurement for worms that had the length and extinction coefficient of adult worms. We confirmed that we were measuring adult animals by asking the sorter to dispense some of those worms and ensuring (by microscopic observation) that the dispensed objects were, in fact, single adult worms.

After pilot experiments (described in S1 Text, Section 6), we generated the following protocol for the rapid measurement of several strains per experiment for the purpose of measuring distributions of whole-animal expression levels.

We collected expression data approximately 24 hours after heat shock. Before measurement, we allowed the worm sorter and laser to warm up for 45 minutes in a small room (5ft x 7ft floor) with a large air conditioner set to 20°. While the room was cooling down and the laser was warming up, we placed 50mL of recovery medium containing the animals into a 50 mL conical tube and allowed the adult animals to sediment by gravity for about five minutes. This gravity sedimentation step allowed us to suck away most of the eggs and hatched L1 larvae from the adult worms. We performed the gravity sedimentation from the original media once and then we washed the animals in 50 mL of sterile S basal and allowed them to sediment again, and removed the supernatant after another five minute sedimentation period. These sedimentation steps eliminated of the vast majority of progeny, while keeping almost all of the adult animals. We then resuspended adult animals in 10-15 mL sterile S-basal in a 50 mL conical tube; sample prep for measurement after heat shock took about 15 minutes at room temperature, which was between 19-23°. We incubated all samples by shaking at 60rpm in 10-15 mL of S-basal at 20° in an incubator in a 20° room; we exercised tight control over temperature at all possible times. We measured animals by putting about 10 mL of S-basal with about 1000 worms, into the sample cup of the Copas Biosort. We then initiated measurement of eGFP or dsRed fluorescence of individual animals.

We kept laser power and PMT gain fixed during measurement of samples expressing the same fluorescent protein. We adjusted PMT gain settings for each experiment to ensure that that the single copy strains were detected (around 30 PMT counts per animal and no zero counts) but that the 530-copy repetitive array animals did not saturate the detector (typically 300-400 PMT counts per animal, but close to 1000 for some animals at the extreme of the population distribution) on a 1024 count scale. Adult animals were typically measured at 7-10 animals per second. Animals were disposed of after measurement.

To prevent cross contamination between measured strains, after running each strain through the instrument, we rinsed out the flow cup three times with approximately 20mL of S-basal, using a large 50mL syringe with about 3cm of tubing attached to the tip to remove the wash fluid. We then ran 10-20 mL of blank S-basal through the sorter between each strain run and initiated the “clean” function, which releases a pulse of high pressure through the tubes through which the worms flow, three times. We ensured that no worms from the prior strain were left in the sample cup or in tubes inside the sorter by attempting to measure blank solution for one minute and detecting no adult-size particles. If we detected any worms in the one minute blank measurement check, which happened only twice in dozens of measurements, we repeated the above procedure, and then did not detect any adults in blank solution, which was cautiously monitored for three minutes after this second cup washing and cleaning procedure.

We conducted two different series of experiments in flow. In one series or "campaign" we measured all of the multicopy reporter strains relative to each other in five different experimental sessions on five different days. In a second campaign, of ten different experimental sessions on ten different days, we measured the lifespan-predicting “reference” multicopy reporter TJ375 (530 Copies) [[58](#_ENREF_58)] and the single copy reporter (TJ3001) [[59](#_ENREF_59)]. Again, each experimental session in each campaign occurred on different days in different weeks with different batches of animals. We measured between two and seven strains in each experiment. Two strains were not present for each experiment in the two campaigns; thus, these strains were not measured as many times as the other reporters. We measured TJ2735 animals in three of the five experiments in the first campaign. We measured TJ3000 animals in three of the ten experiments in the second campaign. We typically measured 500 worms for each strain in each experiment, though we occasionally measured larger and smaller numbers (336-1255); in those cases, the values from these high and low number runs were within the range of runs with 500 animals. Measurement for as many as seven strains took approximately one hour. Experiments comparing expression from the single copy strain, TJ3001, and the multicopy strain, TJ375, took significantly less time (20-30 minutes). In order to avoid possible systematic error introduced by consistently measuring the strains in the same order, and thus at consistently different times (early and late) after heatshock, we varied the order in which we measured the strains, and the order in which we measured reporter color, in each series of measurements

**Measurements of GFP signal recovery after photobleaching:** We mounted animals as described in the main text. In pilot experiments, we determined the total laser power delivered to the sample volume needed for complete photobleaching (i.e., the number of raster scans sufficient to achieve 100% loss of fluorescent signal). We then used a Zeiss LSM 780 with a 488nm laser set to 100% power to photobleach volumes from the nuclei and cytoplasms of individual intestine cells. The confocal optical slice depth was set to one micron and the scanned area was no larger than two square microns. This region of interest was small enough to fit inside the nucleus, permitting us to measure GFP signal before photobleaching and recovered nuclear GFP signal at a single timepoint after photobleaching. We used the Zeiss Zen Black software to control the image-photobleach-image sequence of events. Carrying out this sequence of steps in live animals was sometimes difficult because many animals twitched during imaging (seemingly in reaction to illumination by the photobleaching laser light [[73](#_ENREF_73)]), ruining those measurement attempts. Due to this twitching, we needed to examine more than 30 animals to successfully measure recovered fluorescence in both the cytoplasm and the nucleus of ten cells in three different animals. We then plotted the time-normalized values for the amount of signal not recovered after photobleaching in a boxplot shown in Figure S2.

**Image Cytometry:** We first determined the orientation of the animals in images and then identified individual cells. We describe the identification of cells in the main text, in Results and below, in S1 Text, Section 7. We note that the identification of the cells in the posterior lineage relied on communications from Jim Priess.

As the intestine is anchored anteriorly and posteriorly [[74](#_ENREF_74)], we used the known positions of the anteriormost and posteriormost cells to determine the identity of adjacent cells (see Results, S1 Text, Section 7, Figures S1 and S6). Once we identified a cell, we measured *P_hsp-16.2_-GFP* signal within an equatorial slice of the cell's nucleus (Figures 3 and S7). We used the ImageJ [[75](#_ENREF_75)] ellipse, oval, and selection brush tools to define the perimeter of the nucleus (determined by the human eye) and measured the average of the pixel value inside the perimeter. We repeated these operations (including drawing boundaries) on the image of the nucleus three separate times to determine the error introduced by the human operator, which was around 1% CV. We designated the expression levels as the average of these three measures. For cells that contained two nuclei, we took the average of both. For variably bi-nucleate cells, we always denoted a single nucleus as anterior and left the posterior data field blank. The expression values of different nuclei in the same cell typically differed by less than 8-16%, as expected from being measured at slightly different depths in the intestine cells (Figure S4). We took the average pixel intensity in the nucleus to be a cell’s value because of reasons including but not limited to: 1) some cells have one nucleus and some have two, 2) there is variation in the size of both nuclei and cells, and 3) obtaining a concentration gives us precise information about the quantity of fluorescent protein in a cell even in the face of variation in the area sampled or the total volume of a nucleus or cell (additional reasons presented in Results). We stored quantified expression values for each nucleus in Excel files, together with information about the reporter, specific animal, name of cell and each nucleus, denoted as anterior or posterior. A protocol format for the entire method of adult worm reporter gene quantification in intestine cells, from mounting the animals to analyzing the images, is presented below, in S1 Text, Section 7.

**Statistical analysis:** We used Sigma Stat (Systat Software, Inc., San Jose) to process all *P_hsp16.2_-XFP* expression data. For all datasets, we first determined if values for each dataset were normally distributed using a Shapiro-Wilk normality test. We then used parametric or non-parametric statistics, as appropriate, to determine if there was a significant difference in average expression level or average coefficient of variation (CV; CV = Standard Deviation / Mean)[[76](#_ENREF_76)]. Statistical results are presented in Table S2.

We conducted two campaigns of experiments in flow, described above; thus generating between five and ten measures of population mean and population CV from five to ten independent experiments. The mean and CV datasets were normally distributed for the single copy and repetitive array experiments in series two, so we used a paired t-test to analyze the data to determine if there was a significant difference in the mean and CV of *P_hsp-16.2_-GFP* expression between TJ375 (530 copy) and TJ3001 (single copy). The expression levels for CL2070 and CL2074 were also normally distributed, so we used a paired t-test to determine if there was a significant difference in mean expression for CL2070 and CL2074 (20 and 21 copies). The mean and CV values for the multicopy reporters measured in series one were normally distributed. Thus, we used one-way repeated measures ANOVA followed by a Student-Newman-Keuls test for multiple comparisons to determine if mean or CV of different multicopy strains was significantly different, comparing the five mean or CV values we had for each multicopy strain. We used one-way repeated measures ANOVA followed by a Student-Newman-Keuls test for multiple comparisons to determine if CV of reporter expression from any of the strains was affected by chromosomal location. We used a paired t-test to determine if there was a significant difference in the CV of the two ds-Red *P_hsp-16.2_* reporters, TJ2732 and TJ2733, relative to the other multicopy reporters. We used a Mann-Whitney Rank-Sum Test to determine if 3’UTR, coinjection marker or fluor had a significant effect on the value of CV. We also utilized Two Way ANOVA procedures to test for effects of chromosome, 3’UTR, array type or fluorescent protein on CV for the multicopy reporters.

For image cytometry, we conducted three independent experiments measuring all the intestine cells 8-11 animals for both TJ375 and TJ3001 animals in each experiment. We had between 28 (TJ3001) and 31 (TJ375) values for expression for each cell for each reporter. We used a Two-Way Repeated Measures ANOVA followed by a Holm-Sidak procedure to compare the amount of variation in the expression of the single copy and 530 copy reporters in particular cells.

**Section 6) Pilot experiments to optimize flow measurement and analysis protocols.**

**Determination of developmental synchronization procedure:** We were concerned about potential sources of variation in the standard developmental synchronization procedure (see Materials and Methods) that might possibly contribute to worm-to-worm variation in *P_hsp16.2_-GFP* output.

In the standard synchronization procedure, we obtained eggs by dissolving the adults in alkaline sodium hypochlorite solution and recovering the eggs [[69](#_ENREF_69)]. In a typical procedure, each adult (of which there are hundreds or thousands) contained about a dozen eggs in its uterus, and the embryos in each eggshell were at different stages of development (e.g. 2-cell to > 100-cell embryos). We then allowed all of the recovered eggs to hatch into sterile medium with no food source where they remain arrested as L1 larvae after hatching [[71](#_ENREF_71)]. Animals remain arrested as L1 larvae until they perceive food through the insulin-like signaling system [[70](#_ENREF_70),[72](#_ENREF_72)]. After the previous 16 hour hatchout, we placed the L1 larvae onto NGM media, which allowed the now-synchronized animals to resume development. Because it takes a fertilized egg 12 hours to hatch at 20° [[77](#_ENREF_77)], there is potentially 12 hours between the ages of the first and the last animals to hatch. We therefore tested the effects of: 1) spending different amounts of time between hatching and undergoing the L1 diapause, and 2) spending different amounts of time in the L1 diapause, on worm-to-worm differences in adult *P_hsp16.2_-GFP* reporter expression.

We were also concerned about variation in *P_hsp16.2_-GFP* expression that might have been caused by different population densities of animals in liquid during hatchout. *C. elegans* constitutively secrete pheromones [[78](#_ENREF_78),[79](#_ENREF_79)] that cause L2 to enter an alternative developmental state, dauer [[80](#_ENREF_80)], rather than proceeding into L3. These pheromones also affect the physiology of L1 animals, for example, by inducing expression of *daf-7* [[81](#_ENREF_81)]. To assess possible variation in reporter gene expression due to variable exposure to this pheromone, we tested the possible contribution of different hatchout densities to differences in reporter expression.

We then tested *P_hsp16.2_-GFP* reporter gene expression on five different populations of both TJ375 and TJ3001 animals. The first was synchronized by standard means. We obtained the normally synchronized population using a mixed population of stock hermaphrodites as the starting material from which to extract eggs, followed by hatchout, followed by growth, as described above. The second was the extremely starved L1 population. We obtained it by setting aside L1s from the standard synchronization procedure and allowing them to continue starving for an additional one week at 20°, followed by growth on food to gravid adulthood. The third and fourth populations were "ultrasynchronized". We obtained these as follows. We grew a large population (twenty 10cm NGM plates with 175 animals each) of normally-synchronized animals at 20° until their first eggs were fertilized. These first ovulations occurred at about 54 hours of development from starved L1s on NGM medium with food at 20°. We confirmed that there were only two (or sometimes three) eggs in these animals by examining a dozen test animals under Nomarski DIC optics for the presence of eggs in the uterus. We immediately extracted eggs from these animals with alkaline sodium hypochlorite. Since ovulation and fertilization take about 20 minutes [[82](#_ENREF_82)], all unhatched embryos obtained in this fashion were conceived within about 20 minutes of one another. We placed these animals in hatchout medium at relatively high (1 animal per μL) and relatively low (1 animal per 10 μL) concentration. The fifth population was also ultrasynchronized in the sense that it was derived from eggs all fertilized within the same 20 minute window as above, but, in this case, we allowed the eggs to hatch in the presence of food so the animals did not undergo L1 diapause. We grew these ten sets of animals (five populations of 530 copy TJ375 animals, five of the single copy TJ3001 animals) to gravid adulthood (72 hours at 20°) on five 10cm NGM plates seeded with OP50 (about 875 adults per population).

We then quantified *P_hsp16.2_-GFP* reporter expression, measuring about 500 animals from each of these 10 sets. Compared to the animals synchronized by standard means, none of the other treatments reduced interindividual differences in reporter expression, and interindividual reporter expression was in fact as variable or more variable (Table S3). We concluded that these differences in developmental circumstances (L1 diapause, animal density and exposure to dauer pheromone during hatchout, and amounts of time spent hatched and starving) did not contribute significantly to interindividual variation in adult *P_hsp16.2_-GFP* reporter gene expression. Hence, we decided to use the standard synchronization procedure described in Materials and Methods.

The reason we believe our negative results for this experiment (i.e., that these treatments did not decrease variation), as well as negative results from strain comparison experiments in the main text, is that in studies that subject worm populations to different treatments (not shown), using the same experimental protocols we used here, we have observed consistent (same direction every experiment) differences in worm-to-worm variation, and in fewer experiments (four) than we conducted here. We note that here, our ability to detect differences in worm-to-worm variation in reporter expression between experimental groups is, at the lowest limit so far, an average of a 10% difference in CV. Hence, for our negative results, there is no significant difference in worm-to-worm variation within an empirically determined margin of error of 10%.

**Development of methods to eliminate cross contamination when measuring multiple strains in a single experimental session:** We first performed pilot experiments in which we determined how best to clean the COPAS Biosort instrument to prevent cross-contamination of samples between measurements. To determine a baseline for such contamination, we filled the sample cup with worms (about 1000 adults in 10 mLs), and measured a few hundred worms in flow. We then stopped the instrument, removed the remaining solution from the sample cup with a 50mL syringe tipped with a 3 inch piece of rubber tubing, washed the sample cup once with blank (wormless) S-basal medium, removed the wash solution, added blank medium, and then ran the blank medium through the instrument. After this procedure, there were usually a few animals that appeared in the blank run. These presumably were worms that had remained in the sample cup or had become stuck in the tubes. To clear the machine of these contaminating worms, we eventually learned to wash the flow cup out three times as above and then initiate flow with a blank S-basal medium. During this flow with blank media, we activated the “clean” function in the instrument's software three times. Activating this function caused the machine to push a high pressure pulse of blank medium through the flow system to clean and unclog the tubes. We then ran blank solution for one additional minute without initiating the cleaning function and monitored the efflux to confirm that the cleaning procedure was effective.

**Determination of within-experiment variation, of required sample sizes, and of between-experiment variation:** We wanted to know whether the *P_hsp16.2_-GFP* reporter expression distributions might significantly change in populations cultured in slightly different growth and heat shock microenvironments. We also wanted to know how many animals we needed to sample in a given experiment to describe the population. Would we get similar answers about mean and distribution with different sample sizes? To answer this question, we measured *P_hsp16.2_-GFP* expression from individuals in three different populations of TJ375 animals that had been synchronized from a single parental group, then grown on three different groups of five 10cm NGM Petri dishes seeded with OP50, then heat shocked in different flasks, all as described above. Each population in each flask contained approximately 875 animals in 50 mL of S basal seeded with OP50. From each of the three populations we drew different sample sizes (258, 435 and 632 individuals). We found no difference in mean *P_hsp16.2_-GFP* expression (*P* > 0.6 for all comparisons) and almost identical distributions (Figure S3). From these experiments, we concluded that we were controlling environment and growth conditions sufficiently well within an experiment. We also concluded that approximately 500 animals (give or take a few hundred) was an appropriate sample size, and that samples of animals that numbered a few hundred more or less provided very similar information about the population average and distribution.

To observe if there was significant between-experiment variation in reporter output or instrument function, we measured *P_hsp16.2_-GFP* reporter expression again, in three more synchronized populations of TJ375 animals, in three different experiments, but this time grown on three different days each separated by about a week. We found that the average expression levels on different days were significantly different (331, 453, 531, arbitrary PMT counts; *P* < 0.05; Figure S3) and that the measured CVs differed as well (30%, 18%, 20%). Because of this variation between experiments on different days, to determine if any strain was consistently more or less variable than the other strains, we decided to use statistical methods that took trial to trial variation in account, described in Materials and Methods. Additionally, to be able to compare the amount of variation we observe independently of the mean, both because of these trial effects and because we wanted to compare strains with vastly different means, we decided to use a normalized measure of variation, made popular by [[76](#_ENREF_76)], described further in Materials and Methods.

**Section 7) Detailed protocol for imaging *C. elegans* intestines, identifying intestine cells, and quantifying diffusible fluorescent protein based reporter gene expression in these cells**

**Materials needed (beyond those for general worm husbandry):**

- A point scanning laser confocal microscope (for information on confocal microscopy not covered here see [[83](#_ENREF_83)])
- A *C. elegans* strain that expresses a diffusing fluorescent protein in the gut cells (in the worm literature, a freely diffusing fluorescent protein is often the product of a "transcriptional reporter")
- 1% agarose pads on microscope slides
- 18 x 18 mm microscope cover slips
- Freshly made worm anesthesia solution (0.1% tricaine and 0.01% tetramisole (Sigma Inc.) in M9; be sure to give the compounds plenty of time (more than ten minutes) to dissolve into solution)
- A computer with ImageJ [[75](#_ENREF_75)]

**Step 1: Mounting the animals**

Place a 3-5 microliter drop of worm anesthesia solution on a 1% agarose pad (about 1-2 cm^2^) on a standard glass microscope slide. Mount the animals onto the pad by carefully picking them off of their NGM plates and into the drop of anesthesia on the pad. Allow the worm to swim off the pick into the drop. Carefully pipette another 3-5 microliters of anesthesia onto a glass coverslip (we use 18 x 18mm), and place that coverslip on top of the pad, ensuring that the coverslip first contacts the drop on the coverslip and the worm-containing drop before contacting the agarose pad: doing this decreases the chance that an air bubble will become trapped between the agarose pad and the coverslip. Air bubbles create optical effects that hinder imaging if they are next to an animal.

Allow the anesthesia to take effect for about an hour in a cool moist environment. To do this, place the slides on a plastic platform about 0.5 cm above an ice bath of about 2L of crushed ice inside a plastic box in a 15 C incubator (we used a plastic Tupperware box used for worm cultivation). During imaging, keep the slides not currently being imaged in this box. Temperature on the plastic platform stays at 12-18 C. Temperature on the platform in the box will remain below 20 C while the box is at room temperature for at least 6 hours. The anesthetized animals are on their sides.

**NOTE:** If worms are still moving after 1.5 hours on the slides, the anesthesia was made incorrectly; remake the anesthesia.

**Step 2: Acquiring the images**

For all images, use a 20X 0.8 NA objective. With this objective, there is sufficient resolution to see the nuclei, and the field of view is big enough so that all the intestine cells in adult animals can be captured in two or three fields of view. New researchers should acquire a nonfluorescent image of the animal (either brightfield or Nomarski DIC). New researchers should also, as a positive control for their ability to identify gut cells (via identification of their nuclei), image RBW2 animals (Figure S6; Table 1). These animals express an Emerin-GFP fusion protein which localizes to the inner nuclear envelope making the nuclear boundary easier to see.

To begin imaging, set the image acquisition parameters. First, choose optical filters appropriate for visualization of the particular fluorescent protein being imaged; for GFP we used a 505-550nm bandpass emission filter. Next, set step size in z to two microns; this value determines how much the objective will move in z, and consequently, how much the focal plane will change in z in each step. Then, set the diameter of the pinhole to about one Airy unit in order to get a confocal optical slice thickness of less than two microns (just under the step size in z; additional details in [[83](#_ENREF_83)]). Next set the sampling value; this is the number of samples per pixel and how those samples are treated to generate a value for that pixel. For worm intestine cells, set the sampling value to four samples per pixel and set the pixel output to equal the line average. Then, set the detector gain, laser power and pixel dwell time. The exact values of these settings will vary depending on the sample brightness, optical filters used and the particular laser used, and therefore, are empirically determined for each sample on each microscope. To properly set gain, laser power and pixel dwell time on a particular point scanning confocal, ensure that there is enough signal from the reporter to be minimally twice the intensity above background, and also that the detector is not saturated. Minimize laser power and pixel dwell time in order to minimize physical heating, photochemical damage, and other perturbations to the animals (e.g., neuronal activation of the *lite-1* gene product by blue light [[73](#_ENREF_73)]), but, set the laser power and pixel dwell time so that the animals can be scanned in no longer than three 3-4 minutes per field of view. On the Zeiss LSM510 system used in this study, we used a 535 detector gain, 18% laser power and a 0.80 microsecond pixel dwell time. Next, before finalizing image acquisition parameters, examine a few different worms and ensure that the images generated from those worms contain signal above background and are not saturated.

After setting the image acquisition parameters, begin acquiring images. Plan to image at least ten animals. Program the microscope to take a z stack of each worm to capture sufficient detail in the fluorescence channel to orient the worm (anterior/posterior, dorsal/ventral) and to capture all of the intestine cell nuclei. It is critical to take enough z slices to sufficiently capture all of the intestine cell nuclei present in all the intestine cells in each field of view; otherwise there will be no data for some of the intestine cells. Determine the number of z slices that are sufficient by determining the most objective-proximal and objective-distal portions of the intestine in the field of view and setting the bounds of the z stack to coincide with those closest and farthest points in z that contain intestine tissue. With a 20X objective, this will typically be in the range of 15-30 slices taken with 2 micron steps in z. The intestine cells can be distinguished by the presence of refractile granules under Nomarski DIC optics and by their relatively gargantuan nuclei (the largest nuclei in the adult soma, on the order of 10 x 10 x 12 microns).

Unless the worm is perfectly coiled on the agarose pad, it will be necessary to image it in at least two fields of view. Ensure that there is sufficient overlap between the first and second field of view (and, if needed second and third fields of view) to prevent any confusion when aligning the two or three z stacks that will comprise a single worm later in the process, during image cytometry. Save each z stack as a digital file format compatible with ImageJ. Save the data in whichever file format can retain *all* the metadata from imaging (including physical pixel size, laser power, pixel dwell time, etc.). If “.tif” is an option, and it saves all the metadata, choose .tif. However, software from some microscope manufacturers, such as Zeiss or Leica, does not save all metadata with .tif files. For images captured using equipment from these manufacturers, use the manufacturer-specific file formats, (for Zeiss, ".LSM"; for Leica, “.LEI”) to ensure access all the metadata. Give each file an informative name (e.g., “worm1part1.lsm” and “worm1part2.lsm”) to ensure that images for particular animals will not be mixed up. Later, if the file was not saved as a .tif, use an appropriate ImageJ plugin, such as “lsm reader” to open the file [[75](#_ENREF_75)]. To be able to generate a composite expression profile for the reporter in subsequent image cytometry steps, save files from at least ten animals.

**NOTE:** To better identify the cells in rings V and VI (Step 4 and Figure S6), where the left-handed helical half-twist occurs, less experienced investigators may find it useful to reimage the midsection of the animal at higher laser power to better see the cells.

**Step 3: Determining the orientation of the animal in the images**

To properly identify the intestine cells in the subsequent step, Step 4, it is critical to properly orient the worm in the images. To do so, first, use ImageJ [[75](#_ENREF_75)] to open the z stack files that cover the animal being analyzed. Overlay the individual z stacks together so that the images form the whole worm body.

In these images, the worms are on their sides. Orient the worm using the anatomical features shown in S1. To determine anterior and posterior, first find the pharynx. This relatively large, loosely "violin-shaped" organ, marks the anterior of the animal. The tail and the anus both mark the posterior.

Next, determine dorsal and ventral. Use the vulva as a ventral marker (refer to Figure S1). The visible vulva in any image the worm designates ventral (fluorescent or brightfield; either is fine). In the posterior, use the anus, which also marks ventral. In images without the vulva or anus, use the spermatheca, or the mature oocytes or eggs to designate ventral (Figure S1).

Finally, determine left and right. In the anterior of the animal, the intestine will be on the left side, and the gonad will be on the right. In the posterior of the animal, the gonad will be on the left and the intestine will be on the right, as in Figure S1.

**NOTE:** Once the investigator acquires significant experience, the orientation of the animal can be determined from intestine signal only. To do so, first identify the four mononucleate cells of ring I, discernable by the fact that ring I is the only ring with four cells. This marks the anterior. Next, scanning from anteriormost ring I and proceeding to cells in rings II and III, determine in which row of cells the ring III/ ring IV boundary is most anterior (between an int3 and an int4 cell). That row of cells is the dorsal row (Figure S1, middle panel). Now determine left and right by observing the position of the intestine, relative to the gonad, in z (intestine occupies the left half of the animal in anterior, right in posterior), as indicated above.

**Step 4: Identifying specific intestine cells in oriented microscope images**

*Process overview*: Once the animal is oriented, begin identifying cells in ring I and work posteriorly to ring V. Next, start identifying cells in ring IX and work anteriorly towards ring VI. Use the nuclei to account for the cells; details about the number of cells in each ring and the number of nuclei in each cell type below, and in Results and S1 Text, Section 1. Identify each intestine cell’s nucleus or nuclei by observing a concentrated fluorescent signal in the distinguishingly large nucleus/nuclei in each intestine cell (Figures 3, S1 and S7). RBW2 worms may also be useful to new investigators here (see Step 2).

*Attributes of cells in different rings:* Ring I, the anteriormost ring, has four mononucleate cells. Ring II is composed of two mononucleate cells (although, in rare animals, int2V will undergo an additional nuclear division). Cells in rings III-VII are binucleate; each cell will have an anterior and a posterior nucleus. Cells in rings VIII and IX can be mononucleate or binucleate.

*The left-handed helical half-twist in the middle of the intestine*: The four cells that comprise the helical twist (int5L, int5R, int6L, int6R) are often compressed by the eggs in the uterus and difficult to image, both because of this compression and because of their change in orientation from dorsal ventral to left-right at the very midsection. These circumstances create a thick slice of intestine tissue to image through because cells in rings V and VI are aligned in z (side by side, L/R in the animal). The identities of the cells anterior to the twist are unambiguous because of their clearly dorsal/ventral orientation. Specifically, use the identity of int4V and its posterior connection to int5L to determine the identity of int5L. Use the identity of int4D and its posterior connection to int5R to determine the identity int5R. The identity of the cells posterior to the twist are likewise unambiguous. So, similarly use the identity of int7L and its anterior connection to int6L to identify int6L; and use the identity of int7R and its anterior connection to int6R to identify int6R. Figure S1 shows both a cartoon diagram of the worm intestine and actual images of an animal expressing GFP in its intestine cells. Figure S6 demonstrates the identification of all eight nuclei in the four cells of the intestinal twist in actual microscopic images.

**NOTE:** Make sure to account for all of the nuclei in each cell so that quantification will be easier.

**Step 5: Quantifying reporter expression in images of intestine cells**

Once the cells are identified, finding the z slice in an image stack with the largest diameter image of a particular nucleus is fairly straightforward. Since nuclei are typically 10 X 10 X 12 micron ellipsoids, a typical nucleus can be seen as a bright oval patch in 4-5 2 micron step z slices. To find the equatorial plane of a nucleus in a given cell, scan through the z slices to find the slice in which the nucleus covers the most area, as in the cartoon in Figure 3. Figure S7 shows this process of identifying the equatorial plane of the six nuclei of the six cells in the first two intestine rings (rings I and II) in actual images of an animal’s intestine cells. Note that for cells in ring I, it is often necessary to adjust the contrast and brightness of the image in ImageJ to detect the nuclei in the two cells that are most distant from the objective.

After identifying the equatorial plane of a nucleus, first, use the ImageJ oval, ellipse, and/or selection brush tools to encompass pixels corresponding to the nucleus. It is sometimes necessary to use the selection brush tool to reshape the oval or ellipse shapes because intestinal nuclei in these images sometimes have teardrop or bean or almond shapes. Next, use the “CTRL + M” command to measure the average pixel intensity in the region identified as the nucleus. Examples of shapes of nuclear images are shown in the far right panel of Figure S7. The results of this measurement triggered by CTRL + M will be sent to a results text file window that will open upon the first measurement, as well as additional metadata generated by ImageJ. Copy these values into a spreadsheet along with relevant information like the filename, the z slice, and the name of the cell and, when relevant, whether it was the anterior or posterior nucleus. For mononucleate cells the job ends here. For binucleate cells, such as int3D, measure and record intensity from both int3Da and int3Dp. For binucleate cells, define the cell’s expression level as the average signal from the two nuclei. These signals did not vary more than expected (8-16%) from slight depth effects (shown in Figure S4 and visually apparent in Figure S1) from being in slightly different planes in the intestine cells.

Proceed to quantify reporter expression in all the nuclei of all the cells of the adult hermaphrodite intestine in this fashion. In our hands, for *P_hsp-16.2_* reporters, data from ten animals per strain collected in a single imaging session has been sufficient to establish distinct, consistent cell-specific patterns of expression.

**NOTE:** In the study reported here, in order to diminish measurement error due to manual delineation of the nuclear perimeter with ImageJ, we quantified signal from each nuclear image three different times. After gaining experience in this manual segmentation, we found the CV in the nuclear fluorescence signal from these different measurements was only about 1%. This fact suggests that, once they have gained experience in quantification as above, researchers need to measure nuclear fluorescence from images only once.

**Supporting Information Acknowledgements.**  In addition to researchers named in the main text, we are grateful to Sarah C. Elgin for useful discussions of current understanding of gene silencing and heterochromatin formation.

**References:**

1. Brodsky V, Chernyaev AL, Vasilyeva IA. Variability of the cardiomyocyte ploidy in normal human hearts. Virchows Arch B Cell Pathol Incl Mol Pathol. 1991; 61: 289-294.

2. Guidotti JE, Bregerie O, Robert A, Debey P, Brechot C, Desdouets C. Liver cell polyploidization: a pivotal role for binuclear hepatocytes. J Biol Chem. 2003; 278: 19095-19101.

3. Sulston JE, Horvitz HR. Post-embryonic cell lineages of the nematode, Caenorhabditis elegans. Dev Biol. 1977; 56: 110-156.

4. Hedgecock EM, White JG. Polyploid tissues in the nematode Caenorhabditis elegans. Dev Biol. 1985; 107: 128-133.

5. Lee HO, Davidson JM, Duronio RJ. Endoreplication: polyploidy with purpose. Genes Dev. 2009; 23: 2461-2477.

6. Hirsh D, Oppenheim D, Klass M. Development of the reproductive system of Caenorhabditis elegans. Dev Biol. 1976; 49: 200-219.

7. Leung B, Hermann GJ, Priess JR. Organogenesis of the Caenorhabditis elegans intestine. Dev Biol. 1999; 216: 114-134.

8. Sulston JE, Schierenberg E, White JG, Thomson JN. The embryonic cell lineage of the nematode Caenorhabditis elegans. Dev Biol. 1983; 100: 64-119.

9. Deppe U, Schierenberg E, Cole T, Krieg C, Schmitt D, Yoder B, et al. Cell lineages of the embryo of the nematode Caenorhabditis elegans. Proc Natl Acad Sci U S A. 1978; 75: 376-380.

10. Miller JH, Reznikoff WS, Silverstone AE, Ippen K, Signer ER, Beckwith JR. Fusions of the lac and trp Regions of the Escherichia coli Chromosome. J Bacteriol. 1970; 104: 1273-1279.

11. Horwitz JP, Chua J, Curby RJ, Tomson AJ, Darooge MA, Fisher BE, et al. Substrates for Cytochemical Demonstration of Enzyme Activity. I. Some Substituted 3-Indolyl-Beta-D-Glycopyranosides. J Med Chem. 1964; 7: 574-575.

12. Koch AL. The Role of Permease in Transport. Biochim Biophys Acta. 1964; 79: 177-200.

13. Chalfie M, Tu Y, Euskirchen G, Ward WW, Prasher DC. Green fluorescent protein as a marker for gene expression. Science. 1994; 263: 802-805.

14. Estojak J, Brent R, Golemis EA. Correlation of two-hybrid affinity data with in vitro measurements. Mol Cell Biol. 1995; 15: 5820-5829.

15. Colman-Lerner A, Gordon A, Serra E, Chin T, Resnekov O, Endy D, et al. Regulated cell-to-cell variation in a cell-fate decision system. Nature. 2005; 437: 699-706.

16. Gordon A, Colman-Lerner A, Chin TE, Benjamin KR, Yu RC, Brent R. Single-cell quantification of molecules and rates using open-source microscope-based cytometry. Nat Methods. 2007; 4: 175-181.

17. Bush A, Colman-Lerner A. Quantitative measurement of protein relocalization in live cells. Biophys J. 2013; 104: 727-736.

18. Elowitz MB, Levine AJ, Siggia ED, Swain PS. Stochastic gene expression in a single cell. Science. 2002; 297: 1183-1186.

19. Hirsh D, Cox GN, Kramer JM, Stinchcomb D, Jefferson R. Structure and expression of the collagen genes of C. elegans. Ann N Y Acad Sci. 1985; 460: 163-171.

20. Mello CC, Kramer JM, Stinchcomb D, Ambros V. Efficient gene transfer in C.elegans: extrachromosomal maintenance and integration of transforming sequences. Embo J. 1991; 10: 3959-3970.

21. Fire A. Integrative transformation of Caenorhabditis elegans. EMBO J. 1986; 5: 2673-2680.

22. Fire A, Waterston RH. Proper expression of myosin genes in transgenic nematodes. EMBO J. 1989; 8: 3419-3428.

23. Steiner FA, Henikoff S. Holocentromeres are dispersed point centromeres localized at transcription factor hotspots. Elife. 2014; 3: e02025.

24. Mello C, Fire A. DNA transformation. Methods Cell Biol. 1995; 48: 451-482.

25. Bolten SL, Powell-Abel P, Fischhoff DA, Waterston RH. The sup-7(st5) X gene of Caenorhabditis elegans encodes a tRNATrpUAG amber suppressor. Proc Natl Acad Sci U S A. 1984; 81: 6784-6788.

26. Kelly WG, Xu S, Montgomery MK, Fire A. Distinct requirements for somatic and germline expression of a generally expressed Caernorhabditis elegans gene. Genetics. 1997; 146: 227-238.

27. Praitis V, Casey E, Collar D, Austin J. Creation of low-copy integrated transgenic lines in Caenorhabditis elegans. Genetics. 2001; 157: 1217-1226.

28. Klein TM, Wolf ED, Wu R, Sanford JC. High-velocity microprojectiles for delivering nucleic acids into living cells. Nature. 1987; 327: 70-73.

29. Williams RS, Johnston SA, Riedy M, DeVit MJ, McElligott SG, Sanford JC. Introduction of foreign genes into tissues of living mice by DNA-coated microprojectiles. Proc Natl Acad Sci U S A. 1991; 88: 2726-2730.

30. Plasterk RH, Groenen JT. Targeted alterations of the Caenorhabditis elegans genome by transgene instructed DNA double strand break repair following Tc1 excision. EMBO J. 1992; 11: 287-290.

31. Broverman S, MacMorris M, Blumenthal T. Alteration of Caenorhabditis elegans gene expression by targeted transformation. Proc Natl Acad Sci U S A. 1993; 90: 4359-4363.

32. Berezikov E, Bargmann CI, Plasterk RH. Homologous gene targeting in Caenorhabditis elegans by biolistic transformation. Nucleic Acids Res. 2004; 32: e40.

33. Robert V, Bessereau JL. Targeted engineering of the Caenorhabditis elegans genome following Mos1-triggered chromosomal breaks. EMBO J. 2007; 26: 170-183.

34. Frokjaer-Jensen C, Davis MW, Hopkins CE, Newman BJ, Thummel JM, Olesen SP, et al. Single-copy insertion of transgenes in Caenorhabditis elegans. Nat Genet. 2008; 40: 1375-1383.

35. Frokjaer-Jensen C, Davis MW, Ailion M, Jorgensen EM. Improved Mos1-mediated transgenesis in C. elegans. Nat Methods. 2012; 9: 117-118.

36. Jinek M, Chylinski K, Fonfara I, Hauer M, Doudna JA, Charpentier E. A programmable dual-RNA-guided DNA endonuclease in adaptive bacterial immunity. Science. 2012; 337: 816-821.

37. Dickinson DJ, Ward JD, Reiner DJ, Goldstein B. Engineering the Caenorhabditis elegans genome using Cas9-triggered homologous recombination. Nat Methods. 2013; 10: 1028-1034.

38. Frokjaer-Jensen C. Exciting Prospects for Precise Engineering of Caenorhabditis elegans Genomes with CRISPR/Cas9. Genetics. 2013; 195: 635-642.

39. Kelly WG, Fire A. Chromatin silencing and the maintenance of a functional germline in Caenorhabditis elegans. Development. 1998; 125: 2451-2456.

40. Krause M, Harrison SW, Xu SQ, Chen L, Fire A. Elements regulating cell- and stage-specific expression of the C. elegans MyoD family homolog hlh-1. Dev Biol. 1994; 166: 133-148.

41. Okkema PG, Harrison SW, Plunger V, Aryana A, Fire A. Sequence requirements for myosin gene expression and regulation in Caenorhabditis elegans. Genetics. 1993; 135: 385-404.

42. Yamada K, Tsuchiya J, Iino Y. Mutations in the pqe-1 gene enhance transgene expression in Caenorhabditis elegans. G3 (Bethesda). 2012; 2: 741-751.

43. Pal-Bhadra M, Leibovitch BA, Gandhi SG, Chikka MR, Bhadra U, Birchler JA, et al. Heterochromatic silencing and HP1 localization in Drosophila are dependent on the RNAi machinery. Science. 2004; 303: 669-672.

44. Kanellopoulou C, Muljo SA, Kung AL, Ganesan S, Drapkin R, Jenuwein T, et al. Dicer-deficient mouse embryonic stem cells are defective in differentiation and centromeric silencing. Genes Dev. 2005; 19: 489-501.

45. Knight SW, Bass BL. The role of RNA editing by ADARs in RNAi. Mol Cell. 2002; 10: 809-817.

46. Grishok A, Sinskey JL, Sharp PA. Transcriptional silencing of a transgene by RNAi in the soma of C. elegans. Genes Dev. 2005; 19: 683-696.

47. Grishok A, Pasquinelli AE, Conte D, Li N, Parrish S, Ha I, et al. Genes and mechanisms related to RNA interference regulate expression of the small temporal RNAs that control C. elegans developmental timing. Cell. 2001; 106: 23-34.

48. Knight SW, Bass BL. A role for the RNase III enzyme DCR-1 in RNA interference and germ line development in Caenorhabditis elegans. Science. 2001; 293: 2269-2271.

49. Tabara H, Sarkissian M, Kelly WG, Fleenor J, Grishok A, Timmons L, et al. The rde-1 gene, RNA interference, and transposon silencing in C. elegans. Cell. 1999; 99: 123-132.

50. Loyola A, Tagami H, Bonaldi T, Roche D, Quivy JP, Imhof A, et al. The HP1alpha-CAF1-SetDB1-containing complex provides H3K9me1 for Suv39-mediated K9me3 in pericentric heterochromatin. EMBO Rep. 2009; 10: 769-775.

51. Brower-Toland B, Riddle NC, Jiang H, Huisinga KL, Elgin SC. Multiple SET methyltransferases are required to maintain normal heterochromatin domains in the genome of Drosophila melanogaster. Genetics. 2009; 181: 1303-1319.

52. Towbin BD, Gonzalez-Aguilera C, Sack R, Gaidatzis D, Kalck V, Meister P, et al. Step-wise methylation of histone H3K9 positions heterochromatin at the nuclear periphery. Cell. 2012; 150: 934-947.

53. Tseng RJ, Armstrong KR, Wang X, Chamberlin HM. The bromodomain protein LEX-1 acts with TAM-1 to modulate gene expression in C. elegans. Mol Genet Genomics. 2007; 278: 507-518.

54. Hsieh J, Fire A. Recognition and silencing of repeated DNA. Annu Rev Genet. 2000; 34: 187-204.

55. Hsieh J, Liu J, Kostas SA, Chang C, Sternberg PW, Fire A. The RING finger/B-box factor TAM-1 and a retinoblastoma-like protein LIN-35 modulate context-dependent gene silencing in Caenorhabditis elegans. Genes Dev. 1999; 13: 2958-2970.

56. Kim JK, Gabel HW, Kamath RS, Tewari M, Pasquinelli A, Rual JF, et al. Functional genomic analysis of RNA interference in C. elegans. Science. 2005; 308: 1164-1167.

57. Link CD, Cypser JR, Johnson CJ, Johnson TE. Direct observation of stress response in Caenorhabditis elegans using a reporter transgene. Cell Stress Chaperones. 1999; 4: 235-242.

58. Rea SL, Wu D, Cypser JR, Vaupel JW, Johnson TE. A stress-sensitive reporter predicts longevity in isogenic populations of Caenorhabditis elegans. Nat Genet. 2005; 37: 894-898.

59. Mendenhall AR, Tedesco PM, Taylor LD, Lowe A, Cypser JR, Johnson TE. Expression of a single-copy hsp-16.2 reporter predicts life span. J Gerontol A Biol Sci Med Sci. 2012; 67: 726-733.

60. Bevis BJ, Glick BS. Rapidly maturing variants of the Discosoma red fluorescent protein (DsRed). Nat Biotechnol. 2002; 20: 83-87.

61. Gibson DG. Gene and genome construction in yeast. Curr Protoc Mol Biol. 2011; Chapter 3: Unit3 22.

62. Haithcock E, Dayani Y, Neufeld E, Zahand AJ, Feinstein N, Mattout A, et al. Age-related changes of nuclear architecture in Caenorhabditis elegans. Proc Natl Acad Sci U S A. 2005; 102: 16690-16695.

63. Brenner S. The genetics of Caenorhabditis elegans. Genetics. 1974; 77: 71-94.

64. Stringham EG, Dixon DK, Jones D, Candido EP. Temporal and spatial expression patterns of the small heat shock (hsp16) genes in transgenic Caenorhabditis elegans. Mol Biol Cell. 1992; 3: 221-233.

65. Jones D, Dixon DK, Graham RW, Candido EP. Differential regulation of closely related members of the hsp16 gene family in Caenorhabditis elegans. DNA. 1989; 8: 481-490.

66. Rutledge RG, Cote C. Mathematics of quantitative kinetic PCR and the application of standard curves. Nucleic Acids Res. 2003; 31: e93.

67. Cypser JR, Wu D, Park SK, Ishii T, Tedesco PM, Mendenhall AR, et al. Predicting longevity in C. elegans: Fertility, mobility and gene expression. Mech Ageing Dev. 2013.

68. Schmittgen TD, Livak KJ. Analyzing real-time PCR data by the comparative C(T) method. Nat Protoc. 2008; 3: 1101-1108.

69. Emmons SW, Klass MR, Hirsh D. Analysis of the constancy of DNA sequences during development and evolution of the nematode Caenorhabditis elegans. Proc Natl Acad Sci U S A. 1979; 76: 1333-1337.

70. Vowels JJ, Thomas JH. Genetic analysis of chemosensory control of dauer formation in Caenorhabditis elegans. Genetics. 1992; 130: 105-123.

71. Hong Y, Roy R, Ambros V. Developmental regulation of a cyclin-dependent kinase inhibitor controls postembryonic cell cycle progression in Caenorhabditis elegans. Development. 1998; 125: 3585-3597.

72. Baugh LR, Sternberg PW. DAF-16/FOXO regulates transcription of cki-1/Cip/Kip and repression of lin-4 during C. elegans L1 arrest. Curr Biol. 2006; 16: 780-785.

73. Edwards SL, Charlie NK, Milfort MC, Brown BS, Gravlin CN, Knecht JE, et al. A novel molecular solution for ultraviolet light detection in Caenorhabditis elegans. PLoS Biol. 2008; 6: e198.

74. Vogel BE, Hedgecock EM. Hemicentin, a conserved extracellular member of the immunoglobulin superfamily, organizes epithelial and other cell attachments into oriented line-shaped junctions. Development. 2001; 128: 883-894.

75. Abramoff MD, Magalhaes PJ, Ram SJ. Image Processing with ImageJ. Biophotonics International. 2004; 11: 36-42.

76. Lewontin RC. On the Measure of Relative Variability. Systematic Zoology. 1966; 15: 141-142.

77. Byerly L, Cassada RC, Russell RL. The life cycle of the nematode Caenorhabditis elegans. I. Wild-type growth and reproduction. Dev Biol. 1976; 51: 23-33.

78. Golden JW, Riddle DL. A pheromone influences larval development in the nematode Caenorhabditis elegans. Science. 1982; 218: 578-580.

79. Golden JW, Riddle DL. The Caenorhabditis elegans dauer larva: developmental effects of pheromone, food, and temperature. Dev Biol. 1984; 102: 368-378.

80. Cassada RC, Russell RL. The dauerlarva, a post-embryonic developmental variant of the nematode Caenorhabditis elegans. Dev Biol. 1975; 46: 326-342.

81. Gallo M, Riddle DL. Effects of a Caenorhabditis elegans dauer pheromone ascaroside on physiology and signal transduction pathways. J Chem Ecol. 2009; 35: 272-279.

82. McCarter J, Bartlett B, Dang T, Schedl T. On the control of oocyte meiotic maturation and ovulation in Caenorhabditis elegans. Dev Biol. 1999; 205: 111-128.

83. Handbook of Biological Confocal Microscopy; Pawley JB, editor. New York: Springer. 2006.
